# Synthesis of Imidazolium Cations Linked to Para-t-Butylcalix[4]arene Frameworks and Their Use as Synthons for Nickel-NHC Complexes Tethered to Calix[4]arenes

**DOI:** 10.3390/molecules28155697

**Published:** 2023-07-27

**Authors:** Michael J. Chetcuti, Haithem Naghmouchi, Abdelwaheb Hamdi, Lydia Karmazin

**Affiliations:** 1 Organometallic Chemistry Group, LIMA—UMR CNRS 7042, European School of Chemistry, Polymers and Materials (ECPM), Universities of Strasbourg and of Upper Alsace, 25 Rue Becquerel, 67087 Strasbourg, France; 2 Department of Chemistry, College of Sciences and Arts in Ar Rass, Qassim University, Buraydah 52222, Saudi Arabia; hamdi_chimie@yahoo.fr; 3 LR05ES09 Laboratory of Applied Chemistry and Natural Substances Resources and Environment (LACReSNE), Faculty of Sciences of Bizerte, University of Carthage, Bizerte 7021, Tunisia; 4 Institut Chevreul FR2638, Pôle Diffraction et Diffusion des Rayons X, Cité Scientifique-Université de Lille, Avenue Paul Langevin, CEDEX, 59652 Villeneuve d’Ascq, France

**Keywords:** calixarene, imidazolium salts, nickel N-heterocyclic carbene complexes

## Abstract

A series of cationic p-tert-butylcalix[4]arenes, with side-arms that are functionalized with imidazolium groups, have been synthesized in good yields. The parent tetrahydroxy para-t-butyl-calix[4]arene was dialkylated at the phenolic hydrogen atoms using α,ω-dibromo-alkanes to yield bis(mono-brominated) alkoxy-chains of variable length. The brominated side-arms in these compounds were then further alkylated with substituted imidazoles (N-methylimidazole, N-(2,4,6-trimethyl-phenyl)imidazole, or N-(2,6-di-isopropylphenyl)imidazole) to yield a series of dicationic calixarenes with two imidazolium groups tethered, via different numbers of methylene spacers (*n* = 2–4), to the calixarene moiety. Related tetracationic compounds, which contain four imidazolium units linked to the calix[4]arene backbone, were also prepared. In all of these compounds, the NMR data show that the calixarenes adopted a cone configuration. All molecules were characterized by NMR spectroscopy and by MS studies. Single crystal X-ray diffraction studies were attempted on many mono-crystals of these cations, but significant disorder problems, partly caused by occluded solvent in the lattice, and lack of crystallinity resulting from partial solvent loss, precluded the good resolution of most X-ray structures. Eventually, good structural data were obtained from an unusually disordered single crystal of **5a**, (1,3)-Cone-5,11,17,23-*tetra*-*t*-butyl-25,27-di-hydroxy-26,28-*di*-[2-(N-2,6-diisopropylphenyl-imidazolium)ethoxy]calix[4]arene dibromide and its presumed structure was confirmed. The structure revealed the presence of H-bonded interactions and some evidence of π-stacking. Some of these imidazolium salts were reacted with nickelocene to form the nickel *N*-heterocyclic carbene (NHC) complexes **7a**–**7d**. A bis-carbene nickel complex **8** was also isolated and its structure was established by single crystal X-ray diffraction studies. The structure was disordered and not of high quality, but the structural data corroborated the spectroscopic data.

## 1. Introduction

Both calixarenes and imidazolium salts have been the objects of study by a large number of different research groups in recent years [1,2]. Calixarenes, especially calix[4]arenes, which have been investigated in more detail than other calixarenes, provide a cavity that is tunable in size and in electronic properties, as both the lower and the upper rim of the calixarenes can be functionalized with a wide variety of functional groups. These molecules are under investigation as selective complexing agents and sensors for both neutral and charged ions—more commonly cations [2,3,4,5,6,7] but more recently, anions [8,9,10,11] with applications that include trapping ions, including radioactive or toxic ions [12,13]. Their size-dependent selectivity has been used in the development of catalysts [14,15,16] and has been exploited in organometallic chemistry [17], where unusual reactivity patterns have been observed [18]. Research into their chemistry has been accelerated by synthetic methods that allow the preparation of multi-gram quantities of calixarenes of various sizes in good yield, many of which are now commercially available [19,20].

Imidazolium salts, in particular, those with alkyl chains on the imidazolium nitrogen atoms, are under extensive study as low-melting ionic liquids [21,22] In addition, they are used as starting materials to access N-heterocyclic carbene complexes [23,24,25], which have become ligands of major importance in current organometallic chemistry and homogeneous catalysis [26,27].

A few years ago, we reported that *para*-t-butylcalix[4]arene **1** can be functionalized with alkyne groups to yield a series of molecules with one, two, or four C–C bonds. These species in turn could be reacted with [Co_2_(CO)_8_] to yield a series of cobalt carbonyl species linked to calixarene units via cobalt π-alkyne-bond interactions [28]. We also recently reported on the syntheses and anion-complexing ability of a series of calix[6]arenes that were functionalized with imidazolium ligands [29]. We are still interested in combining the relatively undeveloped organometallic chemistry of calixarenes [30] with our current research on the chemistry of N-heterocyclic carbene complexes of nickel [31,32,33,34,35,36,37,38,39]. This manuscript describes some steps in this direction via the synthesis of a series of calix[4]arene-linked imidazolium cations. Specifically, a series of new cationic *para*-t-butyl-calix[4]arenes, which were functionalized with imidazolium groups, were prepared. These heterocyclic groups were tethered to the calixarene skeleton via variable-length alkyl sidechains. Similar species have already been investigated by various groups in anion-binding studies [40,41,42,43]. The synthesized imidazolium salts that are reported in this manuscript have potential as anion-binding agents, and studies are underway to determine their binding ability and selectivity towards various anions. Indeed, preliminary results show that the ^1^H NMR spectra of the cation of **3c** with PF_6_^−^ as an anion showed significant ^1^H NMR shifts in some peaks. This indicates that the cation interactions with PF_6_^−^ were different here than for those seen for the Br^−^ anion present in **3c** [44].

We also plan to see whether the calixarene-tethered nickel complexes exhibit any advantages (perhaps a cavity effect?) on Suzuki–Miyaura catalysis. We have already reported similar studies with related calix[6]arenes linked to CpNi(NHC) groups and have investigated their catalytic activity, as mentioned earlier [29,30]. Other groups have published examples of catalysis by nickel complexes attached to a supramolecular framework [45,46,47].

## 2. Results and Discussion

**Scheme 1:**
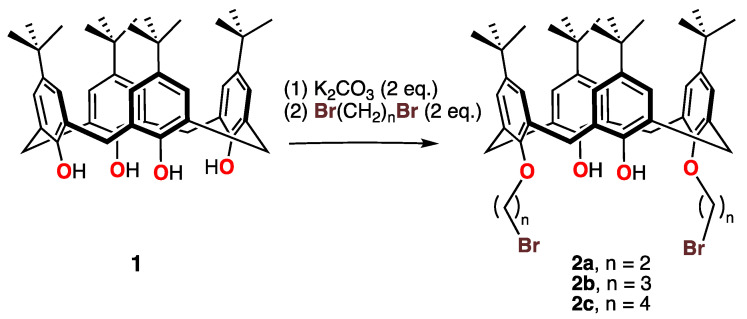
Syntheses of the dibromocalix[4]arenes **2** with various-length methylene spacers.

The reaction of *p*-tert-butylcalix[4]arene **1** with a series of α,ω-dibromoalkanes led to alkylation of two of the calixarene hydroxy groups and to the formation of a series of 1, 3-dialkylated calixarenes with –O(CH_2_)_n_Br groups (**2a**, *n* = 1; **2b**, *n* = 3; **2c**, *n* = 4) in moderate to excellent yields, as shown in Scheme 1.

All reactions were carried out using an excess of α,ω-dibromoalkane. Potassium carbonate, used as a base, was added to an acetonitrile solution of **1** first and stirred for a couple of hours. Then, α,ω-dibromoalkane was introduced, and the mixture refluxed for a period of two days, as shown in the scheme.

After work-up, the series of calixarenes **2**, with brominated side chains on the oxygen atoms in the 1- and 3-positions, were isolated and characterized by ^1^H NMR spectroscopy. The data clearly indicate that the brominated alkyl side chain was added to the calixarene moiety and that the molecule had an effective mirror plane on the ^1^H NMR timescale. Furthermore, the AB patterns and characteristic coupling constant (*J* ≈ 13 Hz) for the methylenic Ar-C*H*_2_-Ar protons and their chemical shifts (≈3.0 ± 2 ppm) indicate that, like the parent calixarene, these bromo-alkylated para-*t*-butylcalixarene derivatives adopted a cone conformation in solution. The presence of the phenolic O*H* protons was confirmed by observation via ^1^H NMR spectroscopy, where they appeared in the 6.6–7.4 ppm range. Detailed ^1^H NMR data for these compounds are listed after the synthesis of each compound.

All compounds were isolated as white or cream-colored solids. It was difficult to obtain them in an analytically pure state, as all the samples trapped solvents, presumably within or close to the calixarene cavity, and these solvents were not easily removed in vacuo. In addition, for probably the same reason, the samples did not crystallize in well-formed mono-crystals, and we were unable to obtain good mono-crystals for a quality single-crystal X-ray diffraction study.

**Scheme 2:**
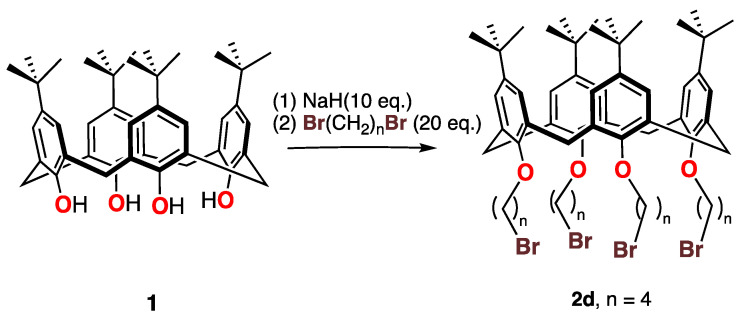
Synthesis of the tetrabromocalix[4]arene **2d**.

By treating the *p*-tert-butylcalix[4]arene **1** with 1-4-dibromobutane and an excess of a stronger base (NaH rather than K_2_CO_3_), the tetra-substituted compound **2d** was obtained in moderate (41%) yield as shown in Scheme 2.The ^1^H NMR spectrum of this compound showed no signals that could be attributed to phenolic protons, as expected.

**Scheme 3:**
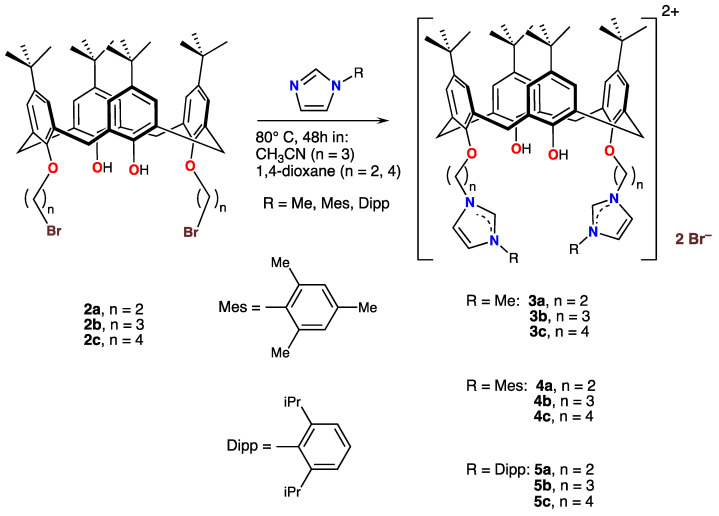
Syntheses and structures of the cationic calix[4]arene imidazolium derivatives **3, 4**, and **5**.

When compounds **2a**–**2c** were treated with N-methylimidazole, N-(2,4,6-trimethylphenyl)imidazole, or N-(2,6-di-isopropyl-phenyl)imidazole ((N-DIPP)imidazole)) in refluxing acetonitrile or in 1,4-dioxane, the unsubstituted nitrogen atom was alkylated: The products were the di-cationic imidazolium salts **3a**–**3c, 4a**–**4c**, and **5a**–**5c**, respectively, as shown in Scheme 3.

All products were obtained as colorless oils or as white or off-white hydroscopic micro-crystalline compounds, which were characterized by ^1^H spectroscopy. The imidazolium proton, on the carbon atom sandwiched between the two imidazolium nitrogen atoms, resonated at ≈ 8 ppm for all complexes. Again, the characteristic C*H*_2_-arene coupling constants of the calix[4]arenes clearly pointed to a cone conformation.

**Scheme 4:**
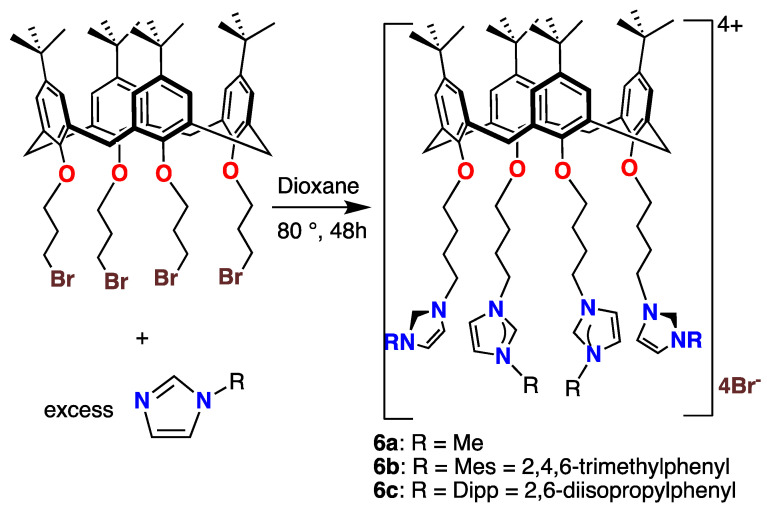
Syntheses and structures of the tetracationic calix[4]arene imidazolium species **6a**–**6c**.

The reactions of the tetrasubstituted calixarene **2d** with the same imidazoles used to prepare compounds **3**–**5** under similar conditions led to the tetraimidazolium tetracationic compounds **6a, 6b**, and **6c** in excellent yields, as shown in Scheme 4. All of these salts were isolated as white or off-white powders. They were soluble in polar solvents but sparingly soluble in pentane or hexane. Most salts exhibited an (M − Br)^+^ m/e signal in their mass spectra (MALDI-TOF, see Procedures). However, in some cases (**6b**, **6c**), a signal for the doubly charged (M − 2Br)^2+^ ion was observed. 

Despite repeated crystallization attempts, none of these salts produced well-formed crystals, which is, unfortunately, not an uncommon occurrence with calixarenes. Single crystals were eventually obtained from two separate samples of **5a**. The data set from one was poor, though it did show the cone configuration of the calixarene.

**Figure 1:**
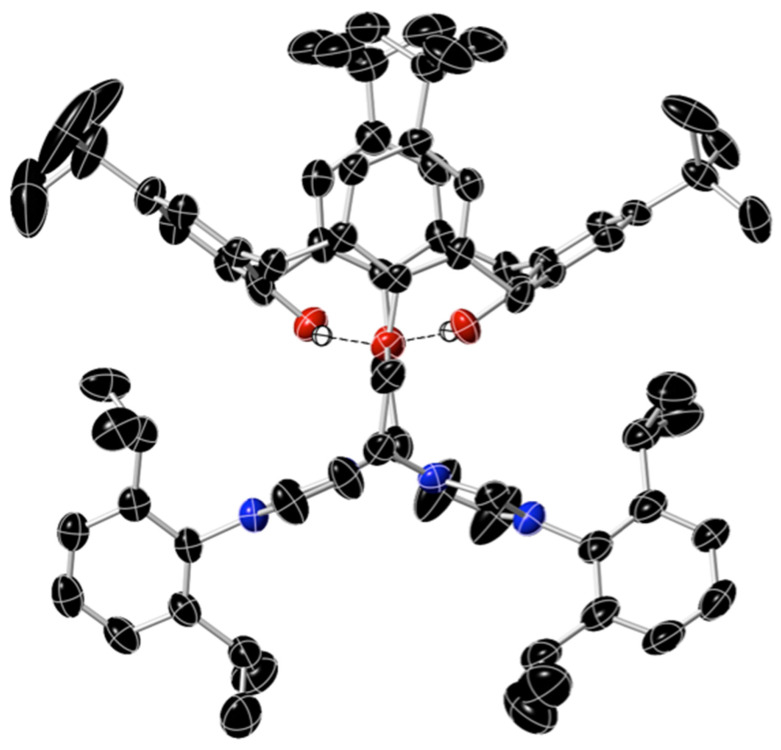
The geometry of the di-cation of **5a**, based on a single crystal X-ray diffraction study. Apart from the hydroxy H-atoms (shown as white spheres), only the atoms of C, O, and N are shown, respectively, as black, red, and blue ellipsoids (at a 50% probability level). One of the four t-Bu groups is rotationally disordered, so only three of the disordered Me carbons are shown around this central t-Bu carbon atom. The H-bonded O^……^H distances are 2.113 and 2.133 Å, whereas the O–H ^……^ O angles are 155.3 and 167.1°.

Another data set from a crystal from the second batch exhibited the same lattice parameters as the previous structural data, but the data here were better, despite the presence of some unusual disorder. The structure was not ideal, but it clearly showed the cone conformation adopted by the di-cationic calixarene and the two substituted imidazolium groups that were linked to the calixarene via a –C_2_H_4_– side chain. One of the bromide anions in this structure was “normal,” but the other had a 50% occupancy of its crystallographic position and shared its lattice location with a methoxy anion (also at 50%). In addition, one of the four t-Bu groups of the calixarene was rotationally disordered. The crystal included H_2_O molecules of crystallization. The structure of the cation of **5a** is shown in Figure 1.

The di-cation had approximate *C*_2_ symmetry in the solid state, as shown. ^1^H NMR data corroborated this cone conformation in solution. Bonds and angles for the calixarene and imidazolium groups were normal. The two calixarene phenoxy rings that contained the OH groups were significantly tilted away from each other, making an angle of 108° to each other. The other two phenoxy rings, linked to the imidazolium groups, were much closer to coplanar, deviating only 4.9° from co-planarity: This indicates the likely presence of π-π interactions between these two rings. This phenomenon was also seen in the two C_6_H_3_iPr_2_ aromatic rings, which exhibited significant π-stacking interactions in the solid state with rings of adjacent molecules, with an arene–arene distance of around 4 Å.

The O^……^O distance for the oxygen atoms of the two ether groups was 5.2 Å, whereas the HO^……^OH distance was 3.17 Å. The phenolic hydrogen atoms were each oriented towards an oxygen atom, and the OH^……^O distances of 2.113 and 2.133 Å indicate significant H-bonding interactions between these two groups in the calixarene ring.

## 3. Synthesis of Nickel NHC Complexes

Our research group has been interested in the chemistry and homogeneous catalysis potential of N-heterocyclic carbene complexes of nickel for the past 15 years, and we have published many papers in this area [31,32,33,34,35,36,37,38,39]. It was of interest to us to graft nickel NHC complexes onto a calixarene backbone to see whether the catalytically active organometallic fragment close to the calixarene cavity would lead to any enhanced catalytic activity. Indeed, we recently described the synthesis of nickel-NHC complexes grafted onto a calix[6]arene framework [29,30]. Thus, we decided to prepare similar complexes in which the metal carbene moiety was linked to a calix[4]arene backbone.

**Scheme 5:**
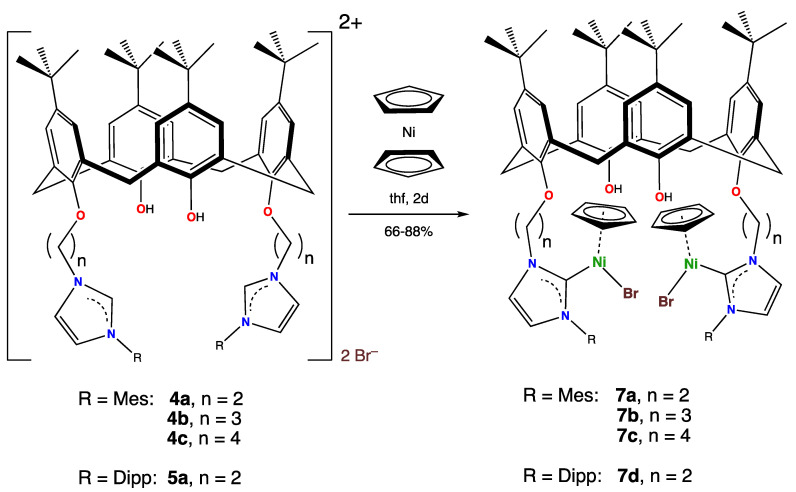
Synthesis of the organometallic complexes **7a**–**7d** from nickelocene and calix[4]arene-bound imidazolium salts.

Imidazolium salts provide a facile entry into nickel NHC chemistry, as the reaction of nickelocene with such species leads to [Ni(NHC)(X)Cp] (X = Cl, Br, I; Cp = η^5^-C_5_H_5_) complexes [23,24,25]. This reaction was carried out with salts **4a**–**4c** and **5a**. When these imidazolium salts were refluxed with two molar equivalents of nickelocene for two days, the bis-carbene complexes **7a**–**d** were obtained in satisfactory isolated yields, ranging from 64–88%, as shown in Scheme 5.

In general, the spectroscopic signatures of the organometallic portions of complexes **7** were similar to those of many other such species that we have reported during our research over the last 15 years. The complexes were all reddish-brown to violet-red in color and exhibited ^1^H NMR spectra that were consistent with their structures. The imidazolium proton on the carbon atom that was sandwiched between the two nitrogen atoms was no longer visible in any of the ^1^H NMR spectra of complexes **7**, as expected, since it was no longer there. The molecule had an effective mirror plane in solution so that only one signal was seen for the η^5^-C_5_H_5_ protons, which appeared (depending on the complex) in the 4.7–4.8 ppm range. The hydroxy protons were observed as singlets at 6.35–6.87 ppm. The AB signals seen for all of these species for the calixarene methylenic protons, with a coupling constant of 13.5–13.9 Hz, indicate that the cone-configuration of the calixarenes, present in the imidazolium salts, was maintained in the organometallic complexes. The MS of complexes **7** exhibited the parent peaks expected for the bis(nickel-NHC) complexes together with the expected isotopic envelope for complexes with two nickel atoms.

**Scheme 6:**
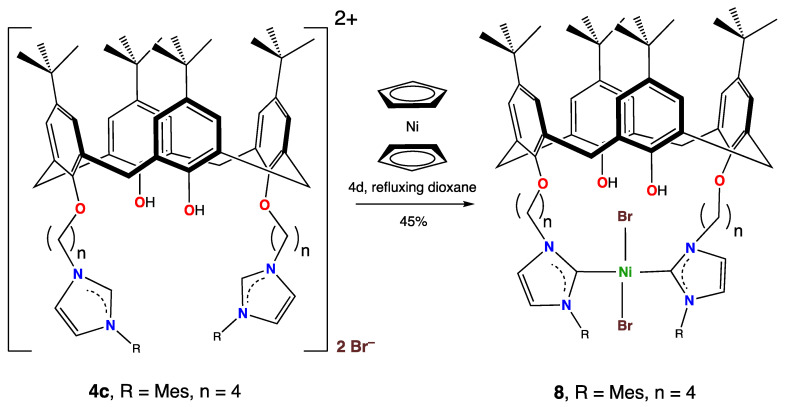
Synthesis of **8** from the reaction of Ni(η-C_5_H_5_)_2_ and **4c** in refluxing dioxane.

When the imidazolium salt **4c** was reacted with Ni(η-C_5_H_5_)_2_ under more forcing conditions (refluxing 1,4-dioxane for 4 days), a different product, **8**, was obtained (Scheme 6) in moderate 45% yield. Its structure was determined via a single crystal X-ray diffraction study, but despite many attempts, only small crystals could be obtained. The resolved structure did not appear to be significantly disordered, apart from commonly observed rotational disorder for some t-Bu groups, but the data were not good due to many weak or absent reflections, and so the esds were high and the structure was not fully refined. Nevertheless, we believe the structure is useful, as it nevertheless established the structure of the compound and yielded further geometric information.

It is quite unusual for an 18-electron organometallic complex to lose a η^5^-C_5_H_5_ ligand, as these ligands are typically inert and not substitutionally labile. A well-known textbook states, “The Cp group […] (is) the most firmly bound … polyenyl and the most inert to nucleophilies or electrophiles” [48]. Although a few examples have been reported [49], they remain unusual.

**Figure 2:**
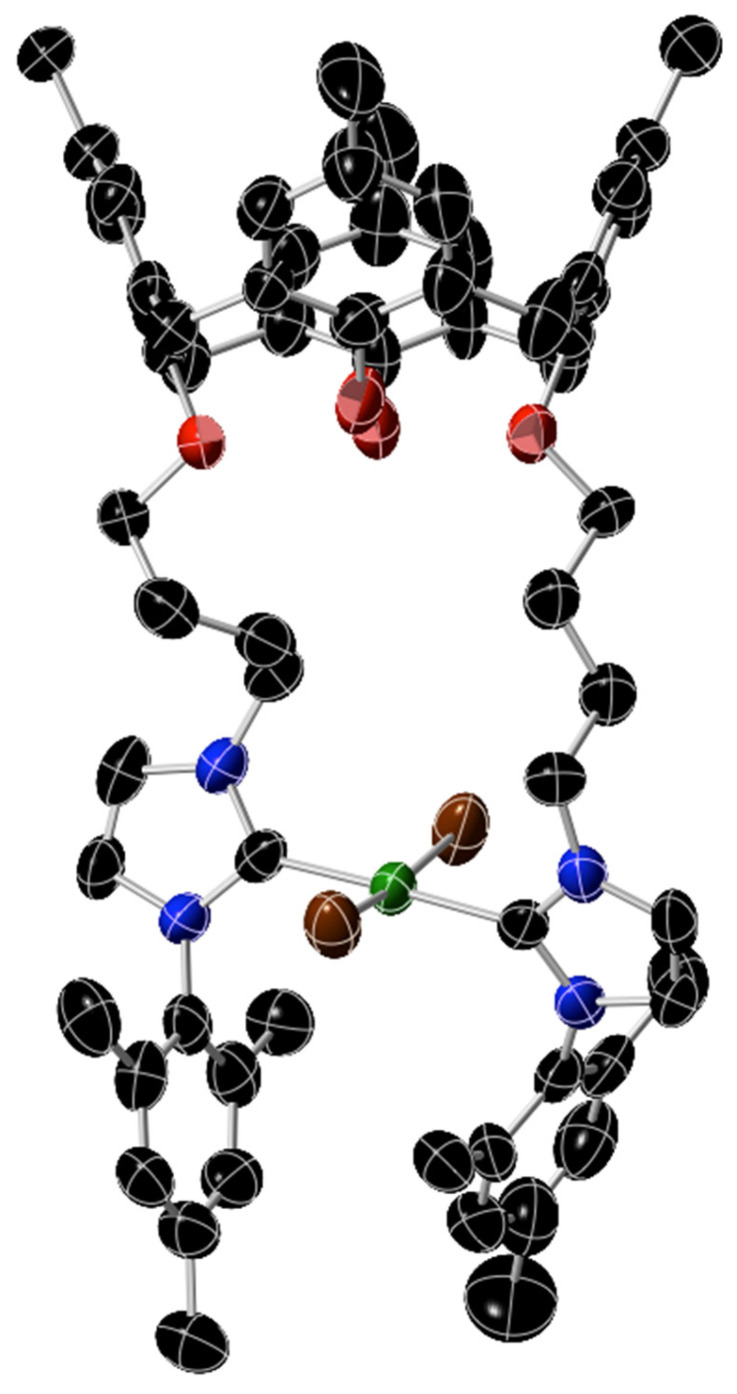
Structure of complex **8**. The atoms are color coded: C, black; O, red; N, blue; Ni, green; Br, brown. The 50% ellipsoids are shown. The structure remains preliminary and is not completely refined as the crystal is small and reflections are weak. Thus, distances and angles are not provided.

The X-ray data show (Figure 2) that **8** is a *bis*-Ni(NHC) complex with no cyclopentadienyl ligands bound to the nickel atom. The nickel was in a *trans*-square-planar geometry with two substituted NHC ligands and two bromides forming the coordination sphere of the metal. The geometry of the complex is analogous to many other complexes of the type *trans*-[NiX_2_(NHC)_2_] (X = Cl, Br, I) that have been reported and have been known for the last 25 years [50,51,52,53]. The coordination of the ligand around the nickel in **8** is broadly similar to what has been observed in such complexes. The calixarene part of this molecule adopted a cone configuration.

The cleavage of Ni–Cp bonds in the formation of **8** is unusual, but other reports of Ni–Cp bond cleavage have been seen for Ni–NHC complexes [54]. Ni–(NHC) bonds decompose in the presence of OH groups and water [55], but this does not appear to have taken place significantly, since it was the Ni–Cp bonds and not the Ni–NHC bonds that were broken in forming complex **8**.

## 4. Conclusions

A series of *bis*-(bromoalkoxy)calix[4]arene derivatives **2a**–**2c** were synthesized by treating the parent p-*t*-butylcalix[4]arene with two equivalents of α,ω-dibromoalkanes and a base (potassium carbonate). A related tetrabromoalkoxycalix[4]arene (**2d**) was also prepared. All of these bromocalix[4]arenes were alkylated further with imidazoles to afford the dicationic imidazolium species **3**–**5** and the related tetracationic imidazolium salts **6**. In all of these species, the imidazolium unit was tethered to the calixarene skeleton by a variable-length methylene spacer (–(CH_2_)_n_, *n* = 2, 4, 6). These salts were fully characterized by ^1^H NMR spectroscopy and by MS, and a single crystal X-ray structural determination was carried out for compound **5a**. Four of these *bis*-imidazolium cations were treated with nickelocene to generate *bis*-(NHC NiCpBr) species (**7a**–**d**) in which the NHC ligands were each linked to the calix[4]arene via (CH_2_)_n_ (*n* = 2–4) spacer groups. Prolonged reaction of **4c** with nickelocene led to unusual loss of the nickel-bound C_5_H_5_ ligand and formed complex **8** with two NHC ligands bound *trans*-in a square planar geometry to a NiBr_2_ unit. The preliminary structure of **8** was determined by an X-ray diffraction study, and it corroborated the spectroscopic data.

Further studies are planned along two research directions. We plan to investigate the Ni–NHC complexes in catalysis with a view to determining whether the calixarene-bound organometallic complexes show any enhanced catalytic effect resulting from the calixarene. Furthermore, as many of the synthesized molecules are di- or tetra-cations, we intend to probe their anion recognition properties and indeed do have some preliminary unpublished results [44].

## 5. Procedures

General. Spectroscopic data are listed for each compound below, but synthetic experimental data are given here only for **2a**, **2d, 3a**, and **7a** (as representative syntheses) since the procedures for the synthesis of the calixarenes with brominated side chains **2a**–**2c**, the imidazolium cations **3**–**6**, and the organometallic nickel NHC complexes **7b**–**7d**, respectively, are similar to each other. Full synthetic data may be found in the Supplementary Material Tables.

All reactions were carried out under an Ar atmosphere. SiO_2_ (Geduran 1.11567) was used for column chromatography. For the calixarene synthesis, reagents (puriss, p. a., grade) were commercial and used without further purification. Solvents were distilled under Ar over sodium/benzophenone (diethyl ether, pentane) or CaH_2_ (dichloromethane) prior to use. DMF (N-N-dimethyl-formamide) was purified by standing over molecular sieves and then was distilled at reduced pressure, and *p*-t-butylcalix[4]arene was prepared following published procedures [19,20]. All ^1^H NMR data were obtained on FT-Bruker Ultra Shield 300 or Spectrospin 400 spectrometers operating at 300.13 or 400.14 MHz, respectively, at room temperature; chemical shifts (∂) are in ppm, relative to residual deuterated solvent peaks in CDCl_3_ (unless otherwise stated). Values of the coupling constant (*J*) are in Hz; peaks are singlets unless otherwise stated; d = doublet, t = triplet, q = quartet, m = multiplet, qn, *J_ap_* = apparent quintet and coupling constant. Mass spectra were recorded on a Bruker micrOTOF-Q mass spectrometer by the Mass Spectroscopy Service (UMR CNRS 7177) at the University of Strasbourg.

X-ray data were collected on a single crystal of **5a** grown from a dichloromethane solution layered with diethylether. Diffraction data were collected at 173(2) K on a Bruker APEX II DUO Kappa-CCD diffractometer equipped with an Oxford Cryosystem liquid N_2_ device using Mo-K*α* radiation (*λ* = 0.71073 Å). The crystal-detector distance was 38 mm. The cell parameters were determined from reflections taken from 3 sets of 12 frames, each at 10 s exposure. The structure was solved using the program SHELXT-2014. The refinement and all further calculations were carried out using SHELXL-2014 [56]. Hydrogen atoms were included in calculated positions and treated as riding atoms using SHELXL default parameters. The non-hydrogen atoms were refined anisotropically using weighted full-matrix least-squares on F^2^. The SQUEEZE instruction in PLATON was applied. The residual electron density was assigned to three molecules of the methanol solvent.

**2a**, (1,3)-cone-5,11,17,23-*tetra*-*t*-butyl-25,27-*di*hydroxy*-bis*(2-bromo-ethoxy)calix[4]-arene.

A mixture of p-*t*-butylcalix[4]arene (3.244 g 5.00 mmol), K_2_CO_3_ (1.383 g, 10.00 mmol), and acetone (50 mL) was stirred for 2 h. Subsequently, a solution of 1,3-dibromopropane (2.834 g, 15.00 mmol) in acetone (50 mL) was added and the mixture was refluxed for 4 d. Methanol (5 mL) was then added, and all solvents were evaporated under vacuum. The residue was dissolved in dichloromethane (100 mL) and extracted with water (150 mL). The organic phase was dried with anhydrous Na_2_SO_4_ and then evaporated to dryness. Washing of the residue with petroleum ether and its subsequent removal afforded **2a**, C_48_H_62_O_4_Br_2_ (3.354 g, 3.887 mmol, 77%), as a fine off-white powder. C_48_H_62_O_4_Br_2_ 862.812 g mol^−1^.

^1^H NMR: 7.062 (4H, Ar–*H*), 6.785 (4H, Ar’–*H*), 6.625 (2H, Ar–O*H*), 4.313 and 3.330 (8H, AB-type doublet, Ar–C*H*_2_–*Ar’*, *J*_AB_ = 13), 4.310 (t, 4H, CH_2_C*H***_2_**O, *J*_HH_ = 6.6), 3.844 (t, 4H, C*H*_2_CH**_2_**O, *J*_HH_ = 6.6), 1.294 (18H, t-*Bu*), 0.950 (18H, t-*Bu*’).

**2b**, (1,3)-Cone-5,11,17,23-*tetra*-*t*-butyl-25,27-*di*hydroxy-26,28-*bis*-(3-bromopropoxy)-calix[4]arene.

^1^H NMR: 7.664 (2H, Ar–O*H*), 7.049 (4H, Ar–*H*), 6.876 (4H, Ar’–*H*), 4.260 and 3.353 (8H, AB-type doublet, Ar–C*H*_2_–*Ar’*, *J*_AB_ = 13), 4.110 (t, *J* = 5.5, 4H, C*H*_2_O,), 4.010 (t, *J* = 6.4, 4H, C*H*_2_Br,), 2.524 (qn, *J*_app_ = 6.1, 4H, CH_2_C*H*_2_CH_2_), 1.273 (18H, t-*Bu*), 1.017 (18H, t-*Bu*’).

**2c**, (1,3)-Cone-5,11,17,23-*tetra*-*t*-butyl-25,27-*di*-hydroxy-26,28-*bis*-(4-bromobutoxy)calix[4]arene dibromide.

^1^H NMR: 7.435 (2H, Ar–O*H*), 7.059 (4H, Ar–*H*), 6.805 (4H, Ar’–*H*), 4.237 and 3.316 (8H, AB doublet, *J*_AB_ = 13, Ar–C*H*_2_–*Ar’*), 4.006 (t, 4H, C*H*_2_O, *J* = 5.8), 3.643 (t, *J* = 6.6, 4H, C*H*_2_Br), 2.335 (qn *J*_ap_ = 4.3, 4H, *CH*_2_*C*H_2_O), 2.162 (qn, *J*_app_ = 4.2, C*H*_2_CH_2_Br), 1.292 (18H, t-*Bu*), 0.967 (18H, t-*Bu*’).

**2d**, (1,3)-Cone-5,11,17,23-*tetra*-*t*-butyl-*tetrakis*(4-bromobutoxy)calix[4]arene.

A mixture of p-*t*-butylcalix[4]arene (7.788g, 12 mmol) and NaH (12 g, 0.5 mmol) was placed in a 500 mL round-bottomed flask under an argon atmosphere, and oxygen and water-free DMF (240 mL) were added. The mixture was stirred for 2 h, and then a solution of dried and freeze-thaw degassed 1,4-dibromobutane (29 mL, 0.240 mol) in DMF (120 mL) was added and the mixture heated under reflux for 4 d. The solvent was then removed under vacuum, and the residue was dissolved in CH_2_Cl_2_ (300 mL) and water 400 mL) was added. The mixture was stirred vigorously and then placed in a large separating funnel, and the organic layer was removed and placed in anhydrous Na_2_SO_4_ to remove any residual water. The dichloroethane solution was then concentrated: The addition of methanol led to the precipitation of **2d** as an off-white powder (C_60_H_84_Br_4_O_4_, 1188,9 g mol^−1^, 5.85 g, 4.92 mmol, 41%).

^1^H NMR: 6.78 (8H, Ar–*H*), 4.35 and 3.13 (AB doublet, *J_AB_* = 12.4, 8H, Ar–C*H*_2_-Ar), 3.91 (t, 8H, C*H*_2_O), 3.51 (t, 8H, C*H*_2_Br), 2.15 (qn, *J_app_* = 7.5, 8H, C*H*_2_CH_2_O), 2.01 (qn, *J_app_* = 7.5, 8H, C*H*_2_CH_2_Br), 0.95 (36H, ^t^*Bu*).

**3a**, (1,3)-Cone-5,11,17,23-*tetra*-*t*-butyl-25,27-*di*-hydroxy-26,28-*bis*-(2-(N-methylimida-zolium)ethoxy)calix[4]arene dibromide.

MS (MALDI TOF): m/z 947.48 (M − Br)^+^.

^1^H NMR: 10.29 (2H, NC*H*N), 8.34 (2H, NC*H*), 7.08 (2H, NC*H’*), 7.26 (4H, Ar-*H*), 6.67 (4H, Ar-*H*), 6.23 (4H, Ar’-*H*), 6.15 (2H, O*H*), 5.20 (t, *J* = 5.0, 4H, CH_2_C*H*_2_O), 4.38 (t, *J* = 5.2, 4H, CH_2_C*H*_2_N), 4.00 (6H, N*Me*), 3.72 and 3.32 (AB doublet, *J*_AB_ = 13, 8H, Ar–C*H*_2_–*Ar’*), 1.33 (18H, t-*Bu*), 0.87 (18H, t-*Bu*’).

**3b**, (1,3)-Cone-5,11,17,23-*tetra*-*t*-butyl-25,27-*di*-hydroxy-26,28-*bis*-(4-(N-methylimida-zolium)propoxy)calix[4]arene dibromide.

MS MALDI TOF, m/z 975.51 (M − Br)^+^.

^1^H NMR: 10.27 (2H, N-C*H*-N), 7.85 (2H, NC*H*-), 7.54 (2H, NC*H’*-), 7.40 (2H, O*H*), 7.07 (4H, Ar-*H*), 6.82 (4H, Ar’-*H*), 4.84 (t, *J* = 7.5, 4H, CH_2_-C*H*_2_-O), 4.12 and 3.36 (AB doublet, *J*_AB_ = 13.4, 8H, Ar–C*H*_2_–Ar’), 4.08 (6H, N-C*H*_3_), 4.07 (t, *J* = 6.3, 4H, CH_2_-C*H*_2_-N), 2.68 (qn, *J*_app_ = 6.6, 4H, CH_2_-C*H*_2_-CH_2_), 1.28 (18H, t-*Bu*), 0.97 (18H t-*Bu’*).

**3c**, (1,3)-Cone-5,11,17,23-*tetra*-*t*-butyl-25,27-*di*-hydroxy-26,28-*bis*-(4-(N-methylimida-zolium)butoxy)calix[4]arene dibromide.

MS (MALDI TOF): m/z 1003.6 (M − Br)^+^.

^1^H NMR: 9.75 (2H, N-C*H*-N), 7.82 (2H, NC*H*-), 7.51 (2H, NC*H’*-), 7.08 (4H, Ar-*H*), 7.00 (2H, O*H*), 6.71 (4H, Ar-*H*), 4.65 (t, *J* = 7.5, 4H, CH_2_-C*H*_2_-O), 4.15 and 3.34 (AB doublet, *J*_AB_ = 13.6, 8H, Ar–C*H*_2_–Ar’), 4.07 (6, N-C*H*_3_), 3.96 (t, *J =* 5.8, 4H, CH_2_-C*H*_2_-N), 2.24 (qn, *J_app_* = 6.8 Hz, 4H, C*H*_2_-CH_2_-O), 1.87 (qn, *J_app_* = 6.3, 4H, *CH*_2_-CH_2_-N), 1.30 (18H, t-*Bu*), 0.88 (18H t-*Bu’*).

**4a**, (1,3)-Cone-5,11,17,23-*tetra*-*t*-butyl-25,27-*di*-hydroxy-26,28-*bis*-[2-(N-2,4,6-trimethyl-phenylimidazolium)ethoxy] calix[4]arene dibromide.

MS (MALDI TOF): m/z 1155.61 (M − Br)^+^.

^1^H NMR: 10.48 (2H, N-C*H*-N), 8.36 (2H, NC*H*-), 7.32 (2H, NC*H’*-), 7.02 (4H, Ar-*H*), 6.94 (4H, Ar’-*H*), 6.57 (4H, Ar’-*H*), 5.86 (2H, O*H*), 5.35 (t, *J* = 4.5, 4H, CH_2_-C*H*_2_-O), 4.37 (t, *J* = 4.35, 4H, CH_2_-C*H*_2_-N), 3.87 and 3.20 (AB doublet, *J*_AB_ = 13.6, 8H, Ar–C*H*_2_–Ar’), 2.34 (6H, Ar-C*H*_3_), 2.00 (12H, Ar-C*H*_3_), 1.30 (18H, t-*Bu*), 0.81 (18H t-*Bu’*).

**4b**, (1,3)-Cone-5,11,17,23-*tetra*-*t*-butyl-25,27-*di*-hydroxy-26,28-*bis*-[3-(N-2,4,6-trimethyl-phenyl)imidazolium)propoxy]calix[4]arene dibromide.

MS (MALDI TOF): m/z 1183.65 (M − Br)^+^.

^1^H NMR: 10.21 (2H, N-C*H*-N), 8.42 (2H, NC*H*-), 7.72 (2H, NC*H’*-), 7.04 (4H, Ar-*H*), 6.97 (4H, Ar-*H*), 6.96 (2H, O*H*), 6.86 (4H, Ar-*H*), 5.29 (t, *J* = 7.5, 4H, CH_2_-C*H*_2_-O), 4.12 (t, *J* = 7.5, 4H, CH_2_-C*H*_2_-N), 4.13 and 3.34 (AB doublet, *J*_AB_ = 13.2, Ar–C*H*_2_–Ar’), 2.79 (qn, *J*_app_ = 6.2, 4H, C*H*_2_CH_2_O), 2.32 (6H, Ar-C*H*_3_), 2.08 (12H, Ar-C*H*_3_), 1.26 (18H, t-*Bu*), 1.01 (18H t-*Bu’*).

**4c**, (1,3)-Cone-5,11,17,23-*tetra*-*t*-butyl-25,27-di-hydroxy-26,28-*di*-[4-(N-2,4,6-trimethylphenyl)imidazolium)butoxy]calix[4]arene dibromide.

MS (MALDI TOF): m/z 1211.58 (M − Br)^+^. M = 1291.42 g.mol^−1^.

^1^H NMR: ^1^H NMR: 10.25 (2H, N-C*H*-N), 8.61 (2H, NC*H*-) 7.16 (4H, NC*H’*- and OH), 6.95 (4H, Ar-*H*), 6.72 (4H, Ar-*H*), 5.12 (t, 4H, *J* = 7.5, CH_2_-C*H*_2_-O), 4.12 and 3.34 (AB doublet, *J*_AB_ = 13.6, 8H, Ar–C*H*_2_–Ar’), 4.07 (t, 4H, *J* = 7.5, CH_2_-C*H*_2_-N), 2.48 (qn *J_ap_* 7.0, 4H C*H*_2_CH_2_O), 2.32 (6H, Ar-C*H*_3_), 2.05 (12H, Ar-C*H*_3_), 1.98 (qn *J_ap_* = 6.4, 4H, C*H*_2_CH_2_N), 1.30 (18H, t-*Bu*), 0.90 (18H t-*Bu’*).

**5a**, (1,3)-Cone-5,11,17,23-*tetra*-*t*-butyl-25,27-di-hydroxy-26,28-*di*-[2-(N-2,6-diisopropyl-phenylimidazolium)ethoxy] calix[4]arene dibromide.

MS (MALDI TOF):, m/z 1239.7 (M − Br).

^1^H NMR: 10.63 (2H, NC*H*N), 8.31 (2H, NC*H*), 7.51 (t, *J* = 7.5, 2H, Ar-*H4*), 7.46 (2H, NC*H’*), 7.28 (d, 4H *J* = 7.5, Ar-H(3), Ar-H(5)), 7.06 (4H, Ar-H), 6.62 (4H, Ar’-*H*), 6.01 (2H, O*H*), 5.46 (t, *J* = 4.4, 4H, CH_2_C*H*_2_O), 4.53 (t, *J* = 4.8, 4H, CH_2_C*H*_2_N), 3.96 and 3.27 (AB doublet, *J*_AB_ = 13, 8H, Ar–C*H*_2_–*Ar’*), 2.26 (m, 4H, C*H*Me_2_), 1.31 (18H, t-*Bu*), 1.19 (d, *J* = 6.9, 6H, CH*Me*_2_), 1.02 (d, *J* = 6.7, 6H, CH*Me*_2_), 0.83 (18H, t-*Bu*’).

**5b**, (1,3)-Cone-5,11,17,23-*tetra*-*t*-butyl-25,27-di-hydroxy-26,28-*bis*-[3-(N-2,6-diisopropyl-phenyl)imidazolium)propoxy]calix[4]arene dibromide.

MS (MALDI TOF): m/z = 1267.73 (M − Br)^+^.

^1^H NMR: 10.35 (2H, NC*H*N), 8.52 (2H, NC*H*), 7.91 (2H, NC*H’*), 7.49 (t, *J* = 4.9, 2H, Ar-*H4*), 7.28 (d, *J* = 4.9, 4H, Ar-H(3), Ar-H(5)), 7.23 (2H, O*H*), 7.02 (4H, Ar-H), 6.89 (4H, Ar’-*H*), 5.44 (t, *J* = 6.5, 4H, CH_2_C*H*_2_O), 4.14 (t, 4H, CH_2_C*H*_2_N), 4.14 and 3.34(AB doublet, *J*_AB_ = 12.6, 8H, Ar–C*H*_2_–*Ar’*), 2.78 (qn, *J_app_* = 6.7, 4H, C*H*_2_CH_2_N), (2.29, m, 4H, C*H*Me_2_), 1.25 (18H, t-*Bu*), 1.18 (d, *J* = 6.7, 6H CH*Me*_2_), 1.15 (d, *J* = 6.7, 6H, CH*Me*_2_), 1.05 (18H, t-*Bu*’).

**5c**, (1,3)-Cone-5,11,17,23-*tetra*-*t*-butyl-25,27-dihydroxy-26,28-*di*-[4-(N-2,6-diisopropyl-phenyl)imidazolium)butoxy]calix[4]arene dibromide.

MS (MALDI TOF): 1295.77 (M − Br)^+^, 100%.

^1^H NMR: 10.31 (2H, NC*H*N), 8.67 (2H, NC*H*), 7.51 (t, 2H, *J* = 8, Ar-*H4*), 7.28(d, 4H, *J* = 8, Ar-H(3), Ar-H(5)), 7.23 (2H, O*H*), 7.15 (2H, NC*H’*), 7.07(4H, Ar-H), 6.76 (4H, Ar’-*H*), 4.19 and 3.37 (AB-type doublet, *J*_AB_ = 13.6, 8H, Ar–C*H*_2_–*Ar’*), 4.12 (t, 4H, *J* = 5.3 CH_2_C*H*_2_N), 4.10 (t, 4H, *J* = 5.3, CH_2_C*H*_2_O), 2.52 (qn, *J* = 7.2, 4H, C*H*_2_-CH_2_-O), 2.30 (m, *J* = 6.6, 4H, C*H*Me_2_), 2.03 (qn *J_ap_* = 5.8, 4H, C*H*_2_CH_2_N), 1.29 (18H, t-*Bu*), 1.20 (d, *J* = 6.6, 6H, CH*Me*_2_), 1.13 (d, *J* = 6.6, 6H, CH*Me*_2_), 0.92 (18H, t-*Bu*).

**6a**, (1,3)-Cone-5,11,17,23-*tetra*-t-butyl-25,26,27,28-*tetra*-[4-(1-N-methylimidazolium)] butoxy-calix[4]arene tetrabromide.

MS (MALDI TOF): m/z 1437.59 (M − Br)^+^.

^1^H NMR: 10.36 (4H, NC*H*N), 8.27 (4H, NC*H*), 7.16 (2H, NC*H’*), 6.77 (4H, Ar-H), 4.72 (t, *J* = 6.9, 8H, CH_2_C*H*_2_O), 4.31 and 3.14 (AB doublet, *J*_AB_ = 14.7, 8H, Ar–C*H*_2_–Ar), 4.00 (12H, N*Me*), 3.94 (t, *J* = 6.5, 8H, CH_2_C*H*_2_N), 2.15 (qn_app_, *J*_app_ = 7, 8H, *CH*_2_CH_2_O), 1.84 (qn_app_, *J*_app_ = 7, 8H, C*H*_2_CH_2_N), 1.07 (36H*, t*-*Bu*).

**6b**, (1,3)-Cone-5,11,17,23-*tetra*(t-butyl)-25,26,27,28-*tetra*(4-N-(2,4,6-trimethylphenyl-imidazolium)butoxy- calix[4]arene tetrabromide.

MS (MALDI TOF): m/z 886.96 (M − 2Br)^+^/2.

^1^H-NMR: 10.34 (4H, N–C*H*–N), 9.02 (4H, N–C*H*), 7.02 (4H N–C*H’*), 6.95 (8H, Ar–*H*), 6.75 (8H, Ar–*H’*), 5.08 (t, *J* = 7.4 Hz, 8H, CH_2_-C*H*_2_-O), 4.35 and 3.13 (AB doublet, *J*_AB_ = 12.4, 8H, Ar-C*H*_2_–Ar), 3.98 (t, 8H, J = 7.4 Hz, CH_2_-C*H*_2_-N), 2.31 (12H, Ar-*pMe*), 2.22 (qn, *J_ap_* = 7.4, *CH*_2_-CH_2_-O), 2.06 (qn*, J_ap_* = 7.4, 8H, C*H*_2_-CH_2_-N), 2.00 (24H, Ar–*oMe*), 1.06 (36H, *t-Bu*).

**6c**, (1,3)-Cone-5,11,17,23-*tetra*(t-butyl)-25,26,27,28-*tetra*-4-N-(2,6-diisopropylphenyl-imidazolium)butoxy- calix[4]arene tetrabromide.

MS (MALDI TOF): 971.06 (M − 2Br)^+^/2.

^1^H NMR: 10.49 (4H, NC*H*N), 9.23 (4H, C*H*N), 7.46 (t, 4H, Ar*H-4*), 7.23 (d, 8H, Ar*H*-3, Ar*H-5)*, 7.03 (4H, C*H*N), 6.75 (8H, Ar-H), 5.16 (t, *J* = 7.0, 8H, CH_2_C*H*_2_O), 4.38 and 3.13, (AB doublet, *J* = 12.8, 8H, Ar-CH_2_-Ar), 4.02 (t, *J* = 7.0, 8H, CH_2_C*H*_2_N), 2.37 (qn_app_, *J* = 7.0, 8H, C*H*_2_CH_2_-O), 2.24 (m, 8, C*H*Me_2_), 2.24 (qn_app_, 8H, *CH*_2_CH_2_N), 1.16 (d, *J* = 6.4, 12H, CH*Me*), 1.10 (d, *J* = 7.1, 12H, CH*Me*), 1.06 (36H, *t*Bu).

### 5.1. Synthesis of **7a**

A mixture of nickelocene (44 mg, 0.232 mmol) and **4a** (143 mg, 0.116 mmol) was placed anaerobically in a Schlenk tube equipped with a reflux condenser, and thf (10 mL) was added. The dark green solution was refluxed for 2 d, and it slowly turned a deep red-violet color during this period. The mixture was cooled and subsequently filtered through a Celite pad, and the solvent was then removed under vacuum. The solid residue was washed with pentane (3 × 10 mL) and again dried under vacuum to afford the NHC complex **7a**, C_82_H_98_N_4_O_4_Br_2_Ni_2__,_ as a red-violet powder (144 mg, 0.0972 mmol, 84%, 1480.9 g mol^−1^).

MS (MALDI TOF): 1401.0 (M − Br)^+^.

^1^H NMR (400 MHz): 8.02 (2H, C*H*-N), 7.08 (4H, Ar-*H*), 7.04 (4H, Ar-*H*), 6.88 (2H, C*H*-N), 6.70 (4H, Ar-*H*), 6.48 (2H, O*H*), 5.61 (t, *J* = 4.5, CH_2_-C*H*_2_-N), 4.77 (10H, *C_5_H_5_*), 4.55 (t, *J* = 4.7, 4H, CH_2_-C*H*_2_-O), 4.11 and 3.32 (AB doublets, *J* = 13.5, Ar-C*H*_2_-Ar), 2.40 (6H, Ar-C*H*_3_), 1.33 (30H, 12 H Ar-C*H*_3_ and 18H, *t-Bu*), 0.87 (18H, *t-Bu*).

**7b**, (C_84_H_102_N_4_O_4_Br_2_Ni_2,_ M = 1508.9 g mol^−1^,110 mg, 73 mmol, 64%).

MS (MALDI TOF) 1429.58 (M − Br)^+^.

^1^H-NMR: 7.66 (2H, C*H*-N), 7.62 (2H, C*H*-N), 7.09 (4H, Ar-*H*), 7.06 (4H, Ar-*H*), 6.89 (2H, O*H*), 6.86 (8H, Ar-*H*), 5.34 (t, 4H, *J* = 6.6*,* CH_2_-C*H*_2_-N), 4.74 (10H, Cp), 4.12 and 3.38 (AB doublets, *J* = 13.2, Ar-C*H*_2_-Ar), 4.12 (t, 4H*, J* = 5.9, CH_2_-C*H*_2_-O), 2.99 (qu, 4H, *J* = 5.8, C*H*_2_-CH_2_-O), 2.41 (6H, Ar-*para*-C*H_3_*), 2.10 (qn, 4H, *J* = 6.3, *CH*_2_-CH_2_-O), 1.30 (12H, br., Ar-*ortho*-C*H_3_*), 1.30 (18H, *t-Bu*), 0.99 (18H, *t-Bu*).

**7c**, (C_86_H_106_N_4_O_4_Br_2_Ni_2_, M = 1536.98 g mol^−1^).

MS (MALDI TOF) 1457.6 (M − Br)^+^, 100%.

^1^H-NMR: 7.52 (2H, C*H*-N), 7.47 (2H, C*H*-N), 7.09 (4H, Ar-*H*), 7.06 4(4H, Ar-*H)*, 6.87 (2H, O*H*), 6.81 (4H, Ar-*H*), 5.18 (t, 4H, *J* = 7*,* CH_2_-C*H*_2_-N), 4.78 (10H, Cp), 4.32 and 3.37, (8H, AB doublets, *J* = 13.5, Ar-C*H*_2_-Ar), 4.12 (t, 4H*, J* = 6.3, CH_2_-C*H*_2_-O), 2.52 (qn, 4H, *J* = 7.0, C*H*_2_-CH_2_-N), 2.41 (6H, Ar-*para*-C*H_3_*), 2.10 (qn, 4H, *J* = 6.3, *CH*_2_-CH_2_-O), 1.55 (12H, br., Ar-*ortho*-C*H_3_*), 1.30 (18H, *t-Bu*), 0.96 (18H, *t-Bu*).

**7d**, C_88_H_110_N_4_O_4_Br_2_Ni_2_, M = 1565 g mol^−1^, 98 mg, 63 mmol, 70%.

MS (MALDI TOF): m/z = 1485.63 (M − Br)^+^, 100%.

^1^H-NMR: 7.90 (2H, C*H*-N), 7.53 (t, 2H, *J* = 7.5, Ar-*H*), 7.35 (d, 4H, Ar-*H*), 7.10 (4H, Ar-*H*), 7.01 (2H, C*H*-N), 6.70 (4H, Ar-*H*), 6.35 (2H, OH), 4.73 (10H, Cp), 4.64 (t, 4H, *J* = 4.8, CH_2_-C*H*_2_-O), 4.09 and 3.31 (8H, AB doublets, *J* = 13.5 Hz, Ar-CH_2_-Ar), 1.86 (m, 4H, *J =* 3.2, C*H*Me_2_), 1.19 (18H, *t-Bu*), 1.19 (d, 6H, *J =* 6.9 Hz, CH*Me*_2_), 0.88 (d, 6H, C*H*Me_2_), 0.88 (18H, *t-Bu*).

### 5.2. Synthesis of Complex **8**

Nickelocene (104 mg, 0.554 mmol), the imidazolium salt **4c** (357 mg, 0.277 mmol), and 1,4-dioxane (10 mL) were introduced into a 50 mL round-bottomed flask equipped with a reflux condenser under Ar, and the mixture was heated, refluxed for 4 d and then allowed to cool. The solution was filtered over Celite, and the solvent was removed under vacuum. The resulting solid was washed with pentane, dried, and then redissolved in toluene. Layering with pentane yielded small crystals of 8, C_76_H_96_Br_2_N_4_O_4_Ni (1348.10 g mol^−1^, 45%, 170 mg, 0.126 mmol).

MS (MALDI TOF): m/z = 1268.20 (M − Br)^+^ 100%.

## Data Availability

Not applicable.

## Supplementary Materials

The following supporting information can be downloaded at: https://www.mdpi.com/article/10.3390/molecules28155697/s1. Synthetic experimental details, 1H NMR data, and the Checkcif file for **5a** are included in the supplementary information tables. Crystal data for complex **5a** has been deposited in the CCDC database as deposition number 2258058 and can be accessed from there. The cif file for 8 is available as supplementary information on request.
