# Supplementary material for: Synthesis of Imidazolium Cations Linked to Para-t-Butylcalix[4]arene Frameworks and Their Use as Synthons for Nickel-NHC Complexes Tethered to Calix[4]arenes
Source: Molecules. 2023 Jul 27;28(15):5697. doi: 10.3390/molecules28155697 (PMC10420804; doi:10.3390/molecules28155697)

Supplementary Materials

Synthesis of Imidazolium Cations Linked to Para-*t*-Butylcalix[4]arene Frameworks and Their Use as Synthons for Nickel-NHC Complexes Tethered to Calix[4]arenes

Michael J. Chetcuti [1,*], Haithem Naghmouchi [1], Abdelwaheb Hamdi [2,3] and Lydia Karmazin [4]

[1] Organometallic Chemistry Group, LIMA—UMR CNRS 7042, European School of Chemistry, Polymers and Materials (ECPM), Universities of Strasbourg and of Upper Alsace, 25 rue Becquerel,
67087 Strasbourg, France; haithem_naghmouchi@yahoo.fr

[2] Department of Chemistry, College of Sciences and Arts in Ar Rass, Qassim University, 52222 Buraydah, Saudi Arabia; hamdi_chimie@yahoo.fr

[3] LR05ES09 Laboratory of Applied Chemistry and Natural Substances Resources and Environment (LACReSNE), Faculty of Sciences of Bizerte, University of Carthage, Bizerte 7021, Tunisia

[4] Institut Chevreul FR2638, Pôle Diffraction et Diffusion des Rayons X, Cité Scientifique - Université de Lille, Avenue Paul Langevin, 59652 Villeneuve d'Ascq CEDEX, France ; lydia.karmazin@univ-lille.fr

* Correspondence: michael.chetcuti@unistra.fr

Pages 2–8	Data and synthetic procedures for compounds not given in the main manuscript
Pages 9–27	^{1}H NMR spectra
Pages 28–31	^{13}C NMR spectra for **3c, 4c**
Page 32	X-ray Data Collection Data
Page 33–39	Check cif for **5a**

Experimental Procedures for the Syntheses of Compounds

General Procedures

All reactions were carried out under an Ar atmosphere. SiO_2 (Geduran 1.11567) was used for column chromatography. For the calixarene synthesis, reagents (puriss, p. a., grade) were commercial and used without further purification. Solvents were distilled under Ar, over sodium/benzophenone (diethyl ether, pentane) or CaH_2 (dichloromethane) prior to use. DMF (N-N-dimethyl-formamide was purified by standing over molecular sieves and then was distilled at reduced pressure, while *p*-t-butylcalix[4]arene was prepared following published procedures. [20,21]. All ^1H NMR data were obtained on FT-Bruker Ultra Shield 300 or Spectrospin 400 spectrometers operating at 300.13 or 400.14 MHz respectively, at room temperature; chemical shifts (∂) are in ppm, relative to residual deuterated solvent peaks in $CDCl_3$ (unless otherwise stated). Values of the coupling constant (J) are in Hz; peaks are singlets unless otherwise stated; d = doublet, t = triplet, q = quartet, m = multiplet, qn, J_{ap} = apparent quintet and coupling constan.

X-ray data were collected on a single crystal of **5a** grown from a dichloromethane solution layered with diethylether. Diffraction data were collected at 173(2) K on a Bruker APEX II DUO Kappa-CCD diffractometer equipped with an Oxford Cryosystem liquid N_2 device, using Mo-Kα radiation (λ = 0.71073 Å). The crystal-detector distance was 38 mm. The cell parameters were determined from reflections taken from three sets of 12 frames, each at 10 s exposure. The refinement and all further calculations were carried out and solved using the program SHELXT-2014 [56]. Hydrogen atoms were included in calculated positions and treated as riding atoms using SHELXL default parameters. The non-hydrogen atoms were refined anisotropically, using weighted full-matrix least-squares on F^2. The SQUEEZE instruction in PLATON was applied. The residual electron density was assigned to three molecules of the methanol solvent.

2a, [(1,3)-cone-5,11,17,23-*tetra-t*-butyl-25,27-*di*hydroxy-*bis*(2-bromo-ethoxy)calix[4]-arene.

A mixture of p-*t*-butylcalix[4]arene (3.244 g 5.00 mmol), K_2CO_3 (1.383 g, 10.00 mmol) and acetone (50 mL) was stirred for 2 h. Subsequently, a solution of 1,3-dibromopropane (2.834 g, 15.00 mmol) in acetone (50 mL) was added and the mixture was refluxed for 4 days. Methanol (5 mL) was then added and all solvents were evaporated under vacuum. The residue was dissolved in dichloromethane (100 mL) and extracted with water (150 mL). The organic phase was dried with anhydrous Na_2SO_4 and then evaporated to dryness. Washing of the residue with petroleum ether and its subsequent removal afforded **2a, $C_{48}H_{62}O_4Br_2$** (3.354 g, 3.887 mmol, 78 %, 826.812 g mol^{-1}) as a fine off-white powder.

2b, [(1,3)-cone-5,11,17,23-*tetra-t*-butyl-25,27-*di*hydroxy-26,28-*bis*-(3-bromopropoxy)-calix[4]arene].

p-*t*-Butylcalix[4]arene (3.244 g, 5.00 mmol) and K_2CO_3 (1.383 g, 10.00 mmol) were placed in a 250 mL round-bottomed flask, and acetone (50 mL) was then added. The mixture was stirred at ambient temperature for 2 h. A solution of 1,4-dibromobutane (3.236 g, 15.00 mmol) in acetone (50 ml) was then added and the reaction mixture was refluxed for 4 d. The solution was then cooled, the solvent was removed under vacuum and the residue was dissolved in dichloromethane (100 mL) and extracted with water (150 mL). The organic phase was dried with anhydrous Na_2SO_4 the solvent was removed. The crude product was washed with pentane to give **2b, $C_{50}H_{66}O_4Br_2$** as a white solid (3.555 g, 3.991 mmol, 80%, 890.865 g mol^{-1}

2c, [(1,3)-Cone-5,11,17,23-*tetra-t*-butyl-25,27-*di*-hydroxy-26,28-*bis*-(4-bromobutoxy)-calix-[4]arene dibromide].

A mixture of *p-tert*-butylcalix[4]arene **1**, (3.244 g, 5.00 mmol) and K_2CO_3 (1.383 g , 10.00 mmol) was placed in a 250 ml round-bottomed flask, and acetone (50 mL) was added. The mixture was stirred for 2 h, and then 1, 4-dibromobutane (3.236 g, 15.00 mmol) was added.

The mixture was refluxed for 4d. Methanol (5 mL) was then added and all the solvents were then evaporated. The residue was dissolved in dichloromethane (100 mL) and washed with water (150 mL). The organic phase was then separated, dried with anhydrous sodium sulfate and concentrated. Addition of pentane precipitated **2c, $C_{52}H_{70}O_4Br_2$** as a white powder (3.834 g, 4.172 mmol, 83%, 918.918 g mol^{-1})

2d, [(1,3Cone-5,11,17,23-*tetra-t*-butyl-*tetrakis*(4-bromobutoxy)calix[4]arene].

p-*t*-Butylcalix[4]arene (7.787 g, 12.00 mmol) and NaH 2.80 g, 118 mmol) were introduced under argon in a 500 mL round bottomed flask. DMF (240 mL) was added, and the slurry stirred for 2 h. 1,4-dibromobutane (28 mL, 240 mmol) in thf (120 mL) was then added and the mixture refluxed for 4 days. All solvents were then removed, and the residue was extracted with dichloromethane (300 mL). The dichloromethane solution of **2d** was then washed with water (400 mL) and the organic phase was separated from the aqueous layer, dried with anhydrous Na_2SO_4 and filtered. Compound **2d** (5.85 g, 4.92 mmol, 41%) was precipitated by the addition of methanol to the dichloromethane solution.

3a, [(1,3)-Cone-5,11,17,23-*tetra-t*-butyl-25,27-*di*-hydroxy-26,28-*bis*-(2-(N-methylimidazolium)-ethoxy)-calix[4]arene] dibromide.

2a (440 mg, 0.511 mmol), 1-methylimidazole (0.326 mL, 4.9 mmol) and 1,4-dioxane (10 mL) were placed under argon in a 50 mL round-bottomed flask equipped with a gas inlet. The mixture was stirred magnetically and was heated to 80 °C for 2d. The solvent was then removed under vacuum, Et_2O (30 mL) was added, and the slurry was stirred for 1 h. The mixture was then filtered to afford **3a**, **$C_{56}H_{74}N_4O_4Br_2$** (385 mg, 0.375 mmol, 73%, 1027.02 g mol^{-1})

3b, [(1,3)-Cone-5,11,17,23-*tetra-t*-butyl-25,27-*di*-hydroxy-26,28-*bis*-(3-(N-methylimidazolium)-propoxy)calix[4]arene] dibromide.

Calixarene **2b** (500 mg, 0.540 mmol), 2,6-di-isopropylphenylimidazole (0.260 mL, 0.032 mmol) and 1,4-dioxane (10 mL) were placed under argon in a 100 mL round bottomed flask equipped with a magnetic stirrer and a gas inlet. The mixture was stirred at reflux for 2d and then the solvent was removed under vacuum. Et_2O (20 mL) was then added and the mixture stirred for 1 h and then filtered to afford **3b, $C_{58}H_{78}O_4N_4Br_2$** (486 mg, 0.461 mmol, 85%, 1055.07 g.mol^{-1} as a pale yellow powder.

3c, [(1,3)-Cone-5,11,17,23-*tetra-t*-butyl-25,27-*di*-hydroxy-26,28-*bis*-(4-(N-methylimida-zolium)butoxy)calix[4]arene] dibromide

2c (850 mg, 0.700 mmol), N-methylimidazole (0.88 mL, 11 mmol) and 1,4-dioxane (10 mL) were placed in a 100 mL round bottomed flask, under argon, stirred magnetically, and heated for 2d at 80 °C. The solvent and excess imidazole were removed under vacuum and Et_2O (20 mL) was added. The mixture was stirred for 1 h and then filtered to give **3c, $C_{60}H_{82}N_4O_4Br_2$** (46 mg, 0.303 mmol, 90%, 1083.13 g mol^{-1}) as a light-yellow powder.

4a, [(1,3)-Cone-5,11,17,23-*tetra-t*-butyl-25,27-*di*-hydroxy-26,28-*bis*-(2-(N-2,4,6-trimethyl-phenylimidazolium)ethoxy)calix[4]arene] dibromide.

2a (400 mg, 0.465 mmol), N-2,4,6-(trimethylphenyl)imidazole (520 mg, 2.0 mmol) and dioxane (10 mL) were placed in a 50 mL round bottomed flask under argon, stirred magnetically, and refluxed for 2d. The solvent was then evaporated, Et_2O (30 mL) was added, and the mixture stirred for 1 h and then filtered to give **4a, $C_{72}H_{90}N_4O_4Br_2$** as a pale-yellow powder in almost good yield (480 mg, 0.389 mmol, 84%, 1235.32 g mol^{-1}).

4b, [(1,3)-Cone-5,11,17,23-*tetra-t*-butyl-25,27-*di*-hydroxy-26,28-*bis*-(3-(N-2,4,6-trimethylphenyl)-imidazolium)propoxy-calix[4]arene] dibromide.

2c (0.400g, 0.450 mmol), N-2,4,6-trimethylphenyl)imidazole (0.366 mL, 3.6 mmol) and acetonitrile (10 mL) were placed in a 100 mL round bottomed flask under argon, stirred magnetically, and refluxed for 2d. The solvent was evaporated, diethylether (30 mL) was

added, the mixture stirred for 1h and then filtered to give **4b, $C_{74}H_{94}N_4O_4Br_2$** (250 mg, 0.200 mmol, 45%, 1263.37 g mol^{-1}) as a pale-yellow powder.

4c, [(1,3)-Cone-5,11,17,23-*tetra-t*-butyl-25,27-di-hydroxy-26,28-*di*-(4-(N-2,4,6-trimethylphenyl)-imidazolium)butoxy-calix[4]arene] dibromide.

2c (0.300g, 0.252 mmol) and (N-2,4,6-trimethylphenyl)imidazole 1.200g, 6 mmol) and 1,4-dioxane (20 mL) were placed in a 100 mL round bottomed flask under argon, stirred magnetically, and heated to 80 °C for 2 d. The solvent was then evaporated and diethylether (30 mL) was added and the mixture stirred for 1h and then filtered to give **4c, $C_{76}H_{98}N_4O_4Br_2$** (302 mg, 0.234 mmol, 93%, 1291.42 g.mol^{-1}) as a pale-yellow powder.

5a, [(1,3)-Cone-5,11,17,23-*tetra-t*-butyl-25,27-di-hydroxy-26,28-*di*-(2-(N-2,6-diisopropylphenyl-imidazolium)ethoxy)calix[4]arene] dibromide.

2a (301 mg, 0.350 mmol), 2,6-diisopropylphenylimidazole (477mg, 2.00 mmol) and acetonitrile (10 mL) were placed in a 50 mL round bottom flask under argon. The reaction mixture was heated to 80 ° and stirred for 2d. The solvent was them removed under vacuum and Et_2O (30 mL) was added and the slurry was stirred for 1h. The product **5a, $C_{78}H_{102}N_4O_4Br_2$** was obtained as a pale-yellow powder by filtration (398 mg, 0.307 mmol, 88%, 1319.48 g mol^{-1}).

5b, [(1,3)-Cone-5,11,17,23-*tetra-t*-butyl-25,27-di-hydroxy-26,28-*bis*-(3-(N-2,6-diisopropylphenyl)-imidazolium)propoxy-calix[4]arene] dibromide.

2b (400 mg, 0.54 mmol), N-(2,6-diisopropylphenyl)imidazole (0.412 mL mg, 3.60 mmol) and acetonitrile (10 mL) were placed under argon in a 100 mL round bottomed flask. The mixture was refluxed at 80 °C for 2d, and then the solvent was removed and Et_2O (30 mL) was added. The mixture was stirred for 1h at ambient temperature. The mixture was then filtered and afforded **5b, $C_{80}H_{106}N_4O_4Br_2$** as a light-yellow powder (414 mg, 0.307 mmol, 67%, 1347.53 g mol^{-1}).

5c, [(1,3)-Cone-5,11,17,23-*tetra-t*-butyl-25,27-dihydroxy-26,28-*di*-(4-(N-2,6-diisopropylphenyl)-imidazolium)butoxy-calix[4]arene] dibromide.

2c (500 mg, 0.55 mmol), N-(2,6-di-isopropylphenyl)imidazole (745 mg, 3.00 mmol) and 1,4-dioxane (10 mL) were placed under argon in a 100 mL round bottomed flask. The mixture was refluxed at 80 °C for 5d, and then the solvent was removed and Et_2O (30 mL) was added. The mixture was refluxed for 1h, and then allowed to stir for 2h at ambient temperature, whilst stirring. The mixture was then filtered and afforded **5c** $\mathbf{C_{82}H_{110}N_4O_4Br_2}$, as a light-yellow powder (650 mg, 0.350 mmol, 86%, 1375.58 g.mol^{-1}).

6a, [cone-5,11,17,23-*tetra*(t-butyl)-25,26,27,28-*tetra* (4-(1-N-methyl)imidazolium) butoxy-calix[4]arene] tetrabromide

The tetrabromo-compound **2d** (400 mg, 0.364 mmol) was added to a 100mL round bottomed flask. 1-Methylimidazole (0.43 mL, 5.3 mmol) and 1,4-dioxane (10 mL) were added and the mixture was magnetically stirred and heated at 80 °C for 2d. The solvent was then removed under vacuum and Et_2O (20 mL) was added. The mixture was stirred, refluxed for 2h, cooled to ambient temperature and then placed in a – 20 °C freezer overnight. The product **6a** was obtained as a pale-yellow powder by filtration (460 mg, 0.303 mmol, 90%, 1517.34 g mol^{-1}).

6b, [cone-5,11,17,23-*tetra*(t-butyl)-25,26,27,28-*tetra*(4-N-(2,4,6-trimethylphenylimidazolium)-butoxy-calix[4]arene] tetrabromide

Compound **2d** (300 mg 0.252 mmol), 2,4,6-trimethyl-phenylimidazole (751 mg, 4.0 mmol) and 1,4-dioxane (10 mL) were added to a 100 mL round bottomed flask equipped with a reflux condenser and inert atmosphere adaptor, and the mixture was heated at 80 °C for 6d and then cooled to ambient temperature. Most of the solvent was removed and diethylether (20 mL) was added. The mixture was then refluxed for 2h, cooled to ambient temperature

and then placed in a -20 °C freezer overnight. **6b**, $\mathbf{C_{108}H_{140}N_8O_4Br_{40}}$ precipitated as a light-yellow powder (454 mg, 0.235 mmol, 93%, 1933.93 g mol^{-1}).

6c, [cone-5,11,17,23-*tetra*(t-butyl)-25,26,27,28-*tetra*(4-N-(2 ,6-diisopropylphenylimidazolium)-butoxy-calix[4]arene] tetrabromide

Compound **2d** (350 mg, 0.294 mmol) and 2,6-diisopropylphenyl-imidazole (1.073 g, 4.67 mmol) and 1,4-dioxane (10 mL) were added to a 100 mL round-bottomed flask, equipped with an argon inlet and a reflux condenser. The mixture was heated with stirring for 6d at 80°C under argon, and the bulk of the solvent was removed under vacuum leaving a pasty solid. Diethylether (20 mL) was then added, the mixture refluxed for 2h, cooled to ambient temperature and then placed in a -20° C freezer. **6c**, $\mathbf{C_{120}H_{164}N_8O_4Br_4}$ (478 mg, 0.227 mmol, 77%, 2102.25 g mol^{-1}) deposited as a pale-yellow powder overnight.

Synthesis of 7a

A mixture of nickelocene (44 mg, 0.232 mmol) and **4a** (143 mg, 0.116 mmol) were placed anaerobically in a Schlenk tube equipped with a reflux condenser, and thf (10 mL) was added. The dark green solution was refluxed for 2d, and it slowly turned a deep red-violet color during this period. The mixture was cooled and subsequently filtered through a Celite pad, and the solvent was then removed under vacuum. The solid residue was washed with pentane (3 x 10 mL) and again dried under vacuum to afford the NHC complex **7a,** $\mathbf{C_{82}H_{98}N_4O_4Br_2Ni_2}$ as a red-violet powder (144 mg, 0.0972 mmol, 84%, 1480.9 g mol^{-1}).

Synthesis of 7b

A mixture of nickelocene (51 mg, 0.270 mmol) and **4b** (168 mg, 0.133 mmol) were placed anaerobically in a Schlenk tube equipped with a reflux condenser, and thf (10 mL) was added. The solution was refluxed for 2d, and it slowly turned a deep red-violet color during this period. The mixture was cooled and subsequently filtered through a Celite pad, and the

solvent was then removed under vacuum. The solid residue was washed with pentane (3 x 10 mL) and again dried under vacuum to afford the NHC complex **7b** as a red-violet powder (**$C_{84}H_{102}N_4O_4Br_2Ni_2$**, M= 1508.9 g mol^{-1}, 110 mg, 0.073 mmol, 55 %).

Synthesis of 7c

Nickelocene (104 mg, 0.554 mmol), and **4c** (357 mg (0.276 mmol) were added to a Schlenk tube equipped with a reflux condenser and 10 mL of THF were added. The mixture was then refluxed anaerobically for 2d and it slowly turned red-violet in color. It was then allowed to cool to ambient temperature. The resulting mixture was passed through a Celite column, and the column was washed with more THF till the filtrate was colorless. The solvent was then removed in vacuum and the residue was washed repeatedly with pendant and then dried to give **7c** (**$C_{86}H_{106}N_4O_4Br_2Ni_2$**, M = 1536.98 g.mol^{-1}, 330 mg,0.216 mmol, 78%).

Synthesis of 7d.

Nickelocene (37 mg, 0.195 mmol), **5a** (120 mg, 0.0909 mmol) and thf (10 mL) were introduced into a small Schlenk tube equipped with a reflux condenser and argon inlet, and the mixture was refluxed for 2d. The mixture was then cooled and filtered through a Celite pad till the washings were clear. The solvent was then removed, and the residue was washed with pentane and dried under vacuum to give **7d** as a reddish-brown powder (**$C_{88}H_{110}N_4O_4Br_2Ni_2$**, M= 1565.03 g mol^{-1}, 98 mg, 0.063 mmol, 69%).

^{1}H NMR spectrum of 2a

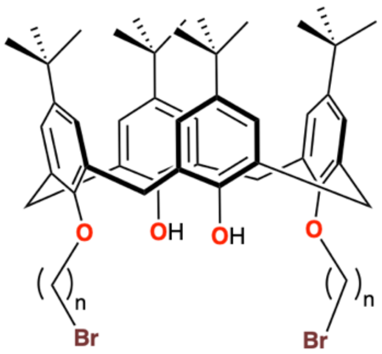

2a, n = 2

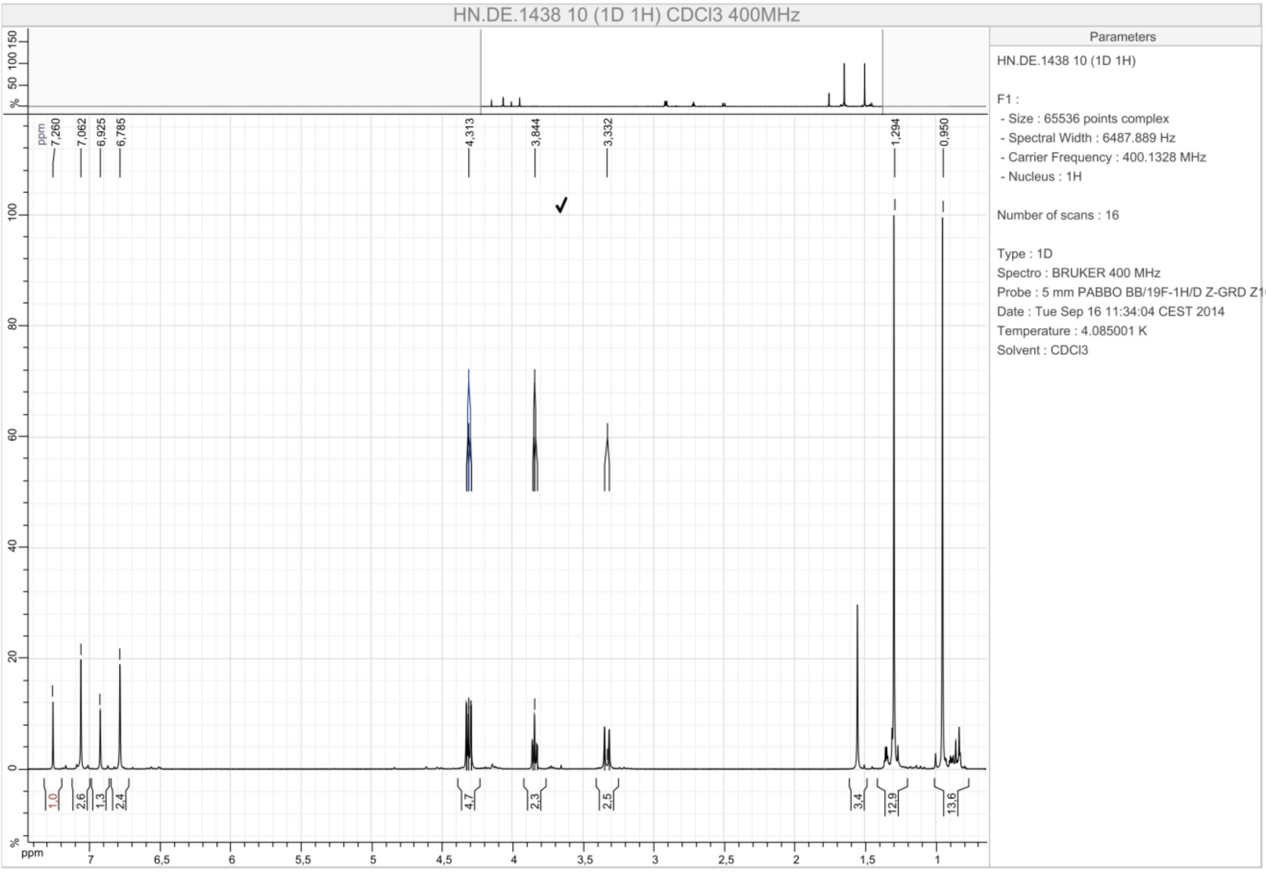

^{1}H NMR spectrum of 2b

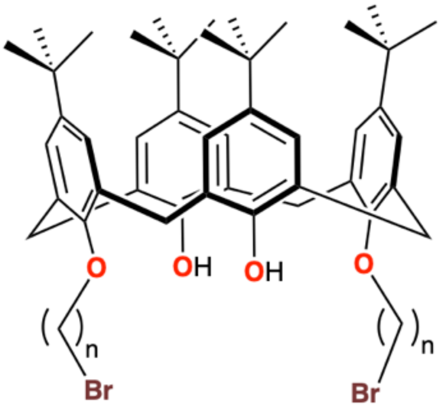

2b, n = 3

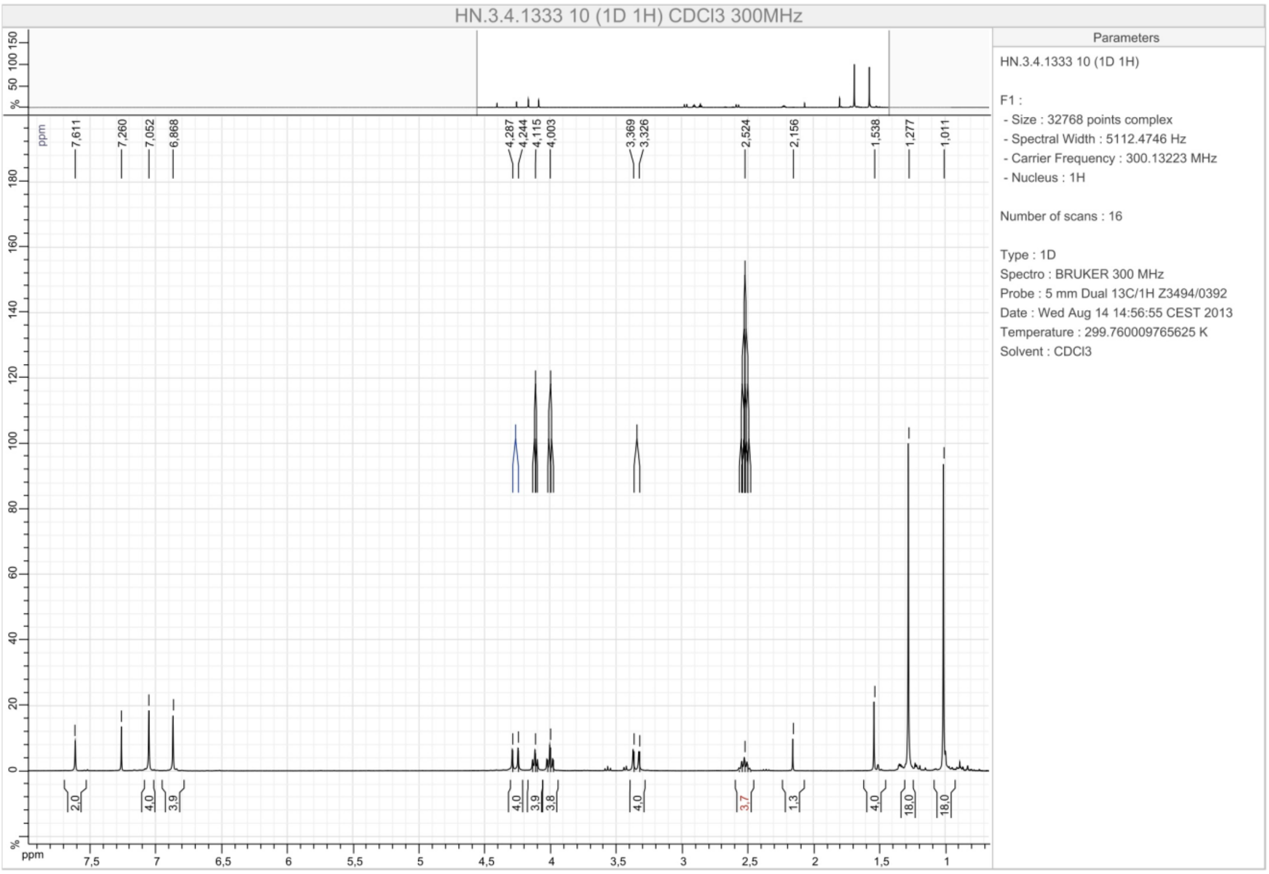

^{1}H NMR spectrum of 2c

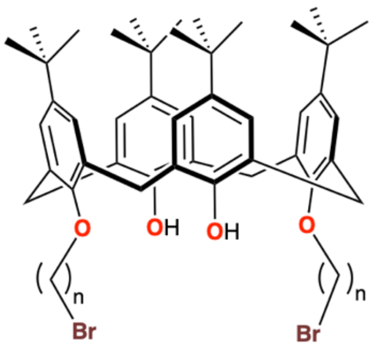

2c, n = 4

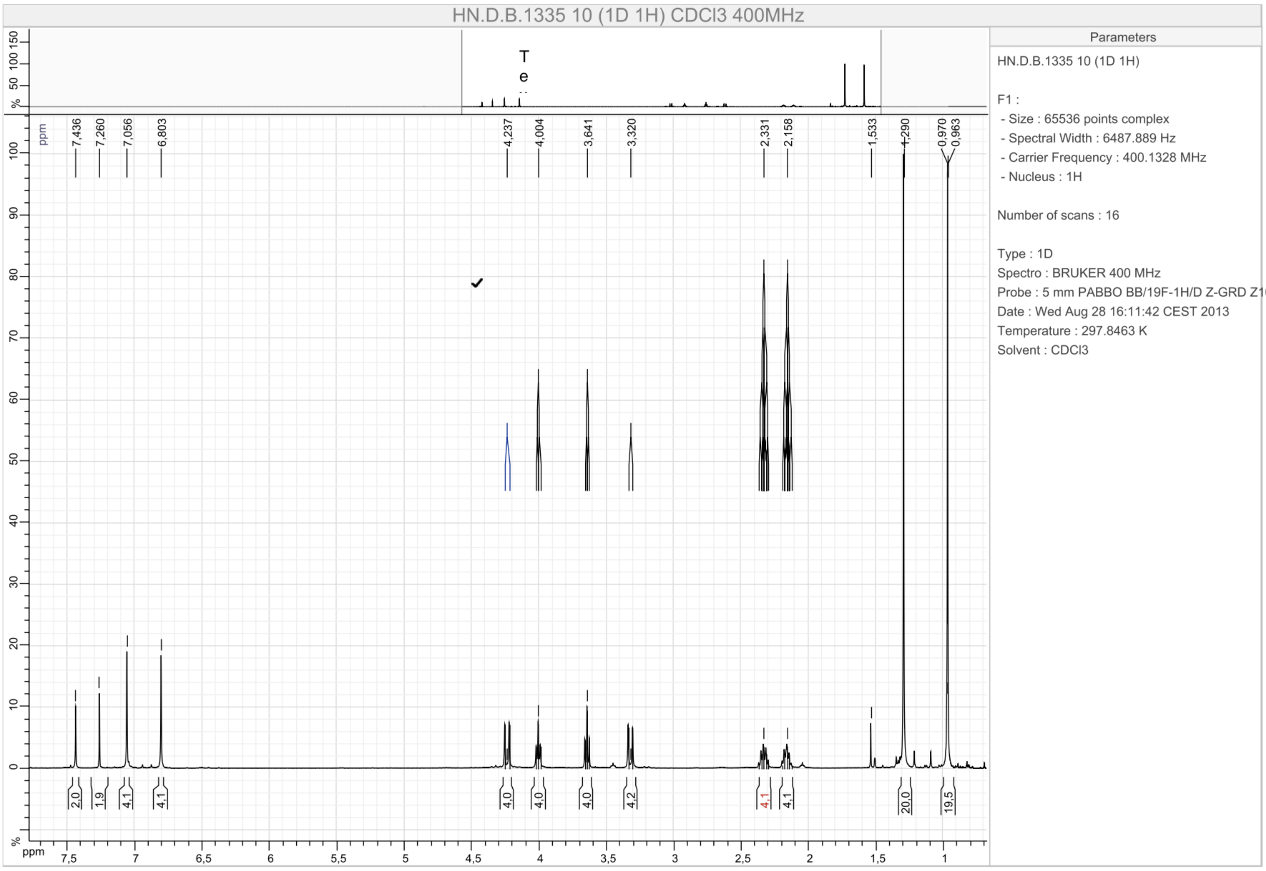

^{1}H NMR spectrum of 2d

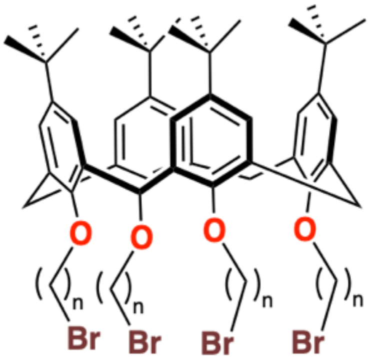

2d, n = 4

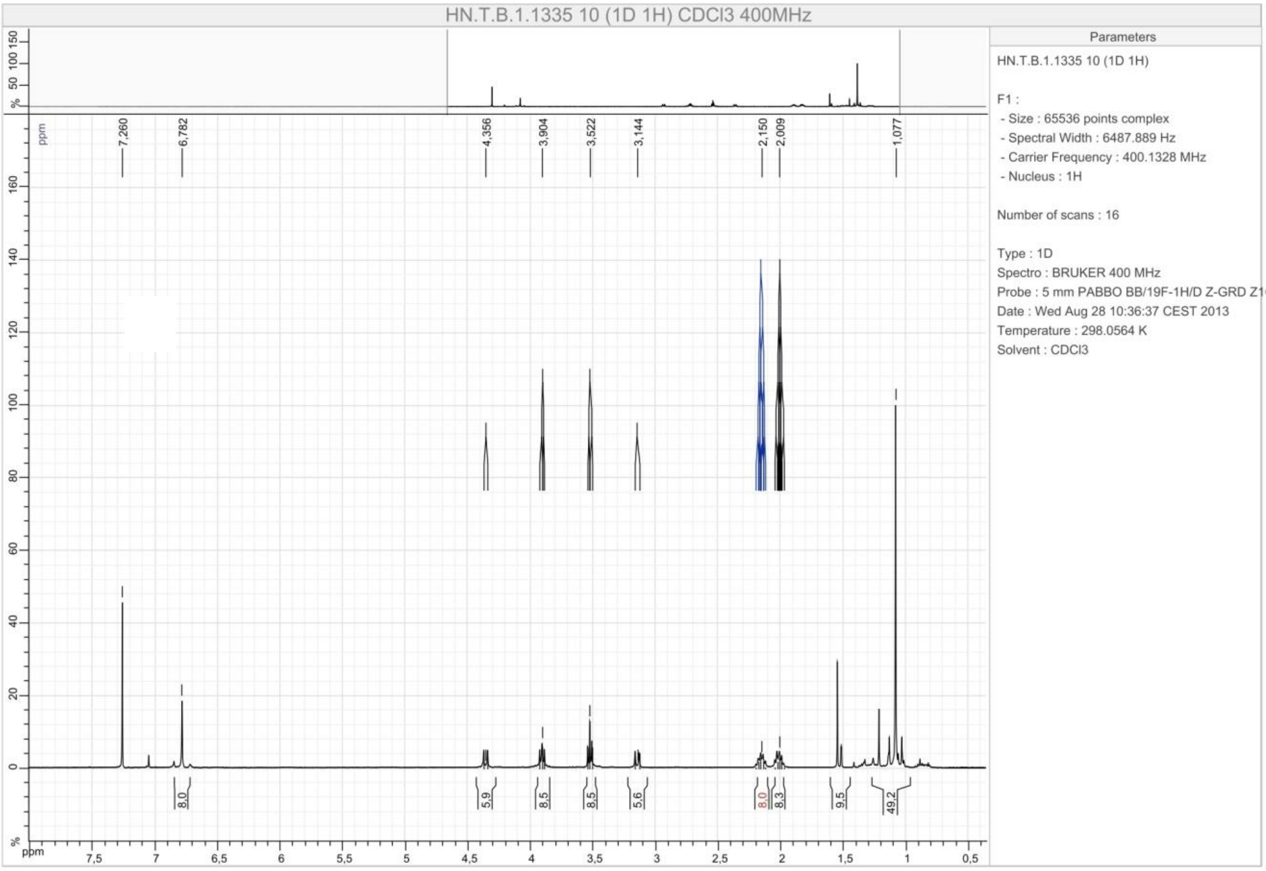

^{1}H NMR spectrum of 3a

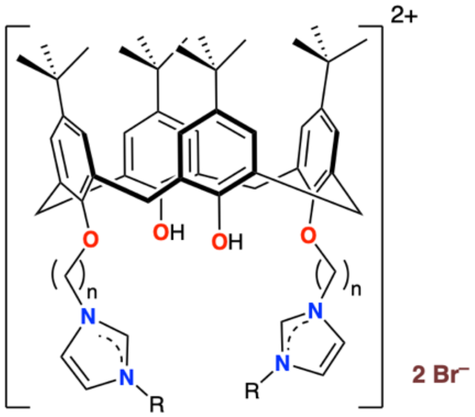

3a, R = Me, n = 2

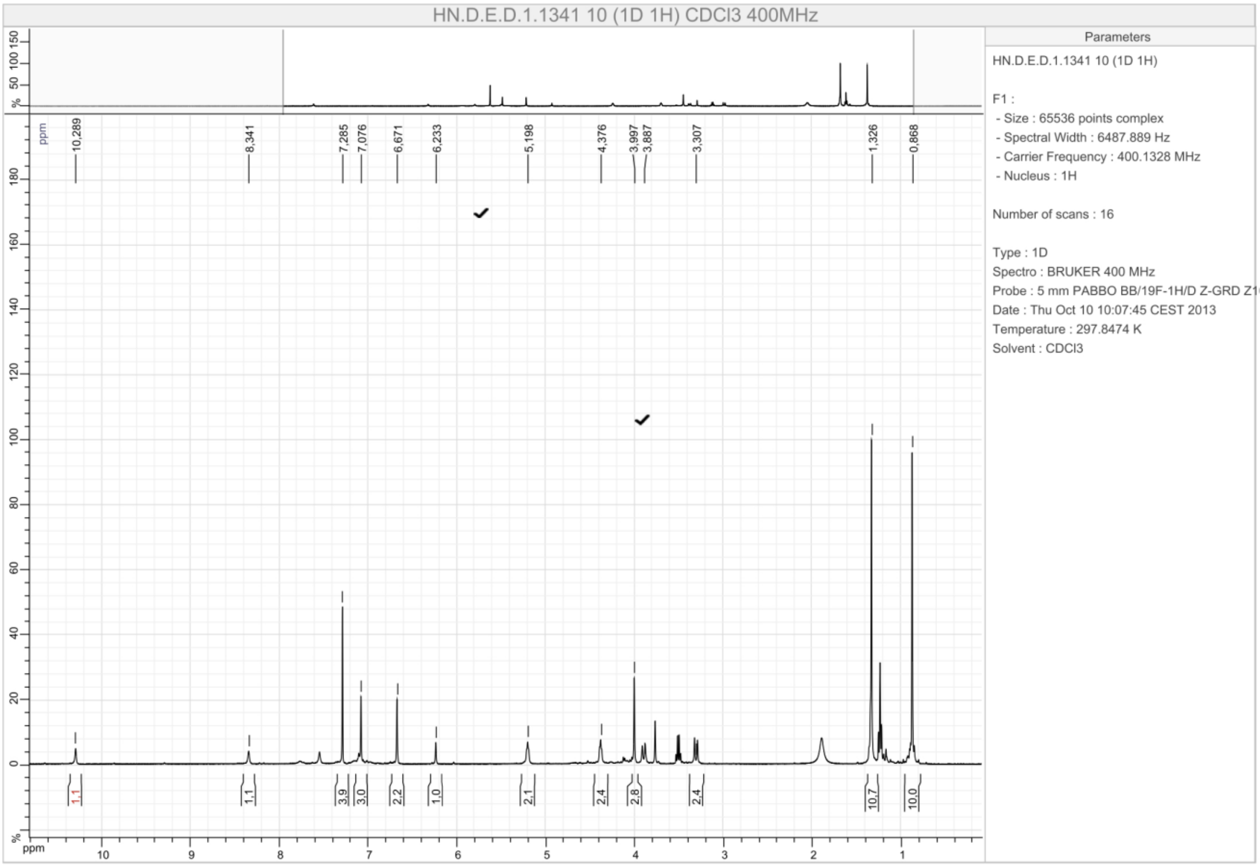
HN.D.E.D.1.1341 10 (1D 1H) CDCl3 400MHz
Parameters
HN.D.E.D.1.1341 10 (1D 1H)
F1 :
- Size : 65536 points complex
- Spectral Width : 6487.889 Hz
- Carrier Frequency : 400.1328 MHz
- Nucleus : 1H
Number of scans : 16
Type : 1D
Spectro : BRUKER 400 MHz
Probe : 5 mm PABBO BB/19F-1H/D Z-GRD Z1
Date : Thu Oct 10 10:07:45 CEST 2013
Temperature : 297.8474 K
Solvent : CDCl3
10,289
8,341
7,285
7,076
6,671
6,233
5,198
4,376
3,997
3,887
3,307
1,326
0,868
1,1
1,1
3,9
3,0
2,2
1,0
2,1
2,4
2,8
2,4
10,7
10,0
ppm

^{1}H NMR spectrum of 3b

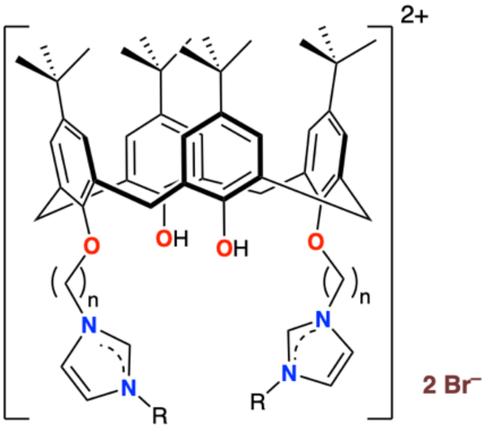

3b, R = Me, n = 3

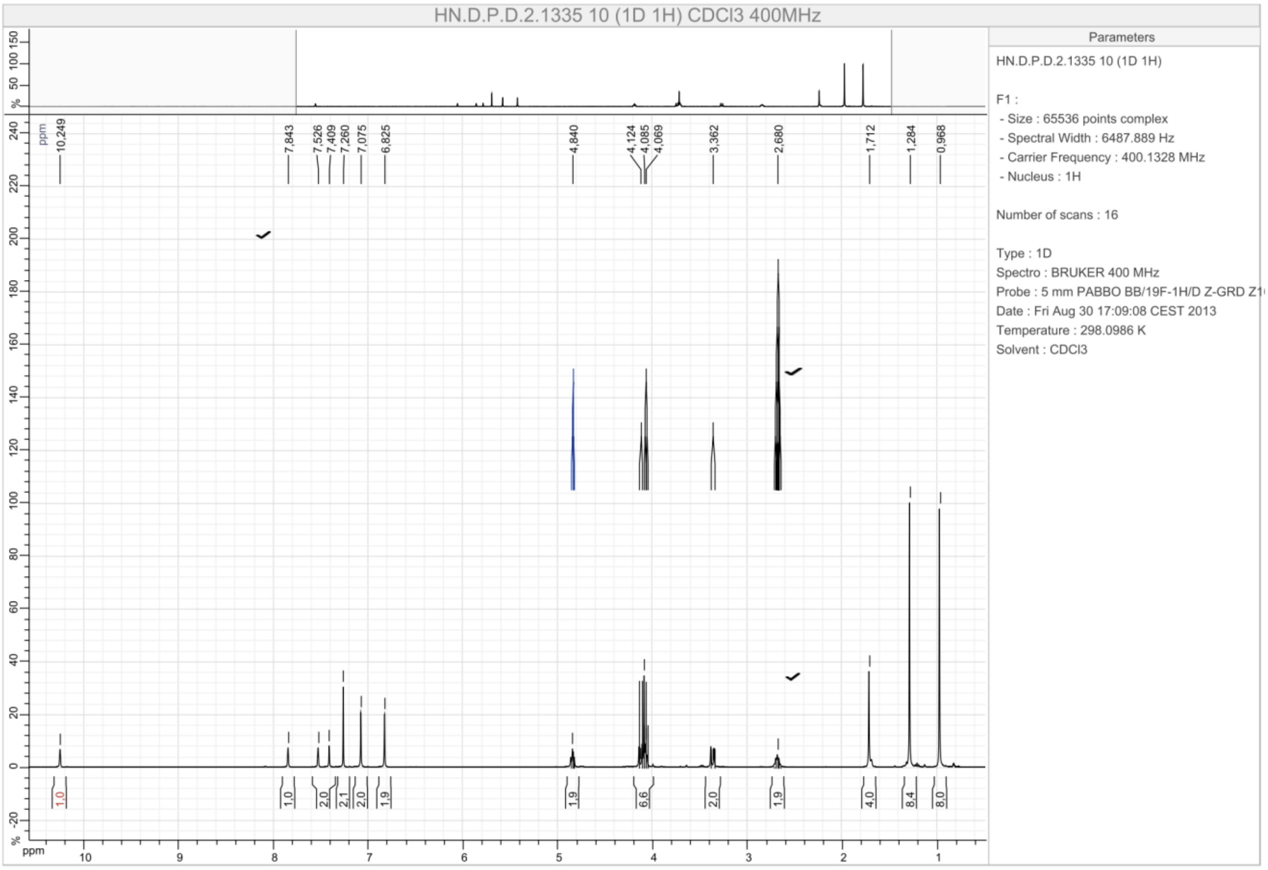

^{1}H NMR spectrum of 3c

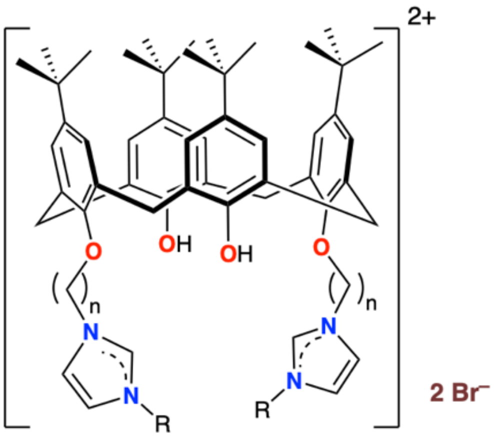

3c, R = Me, n = 4

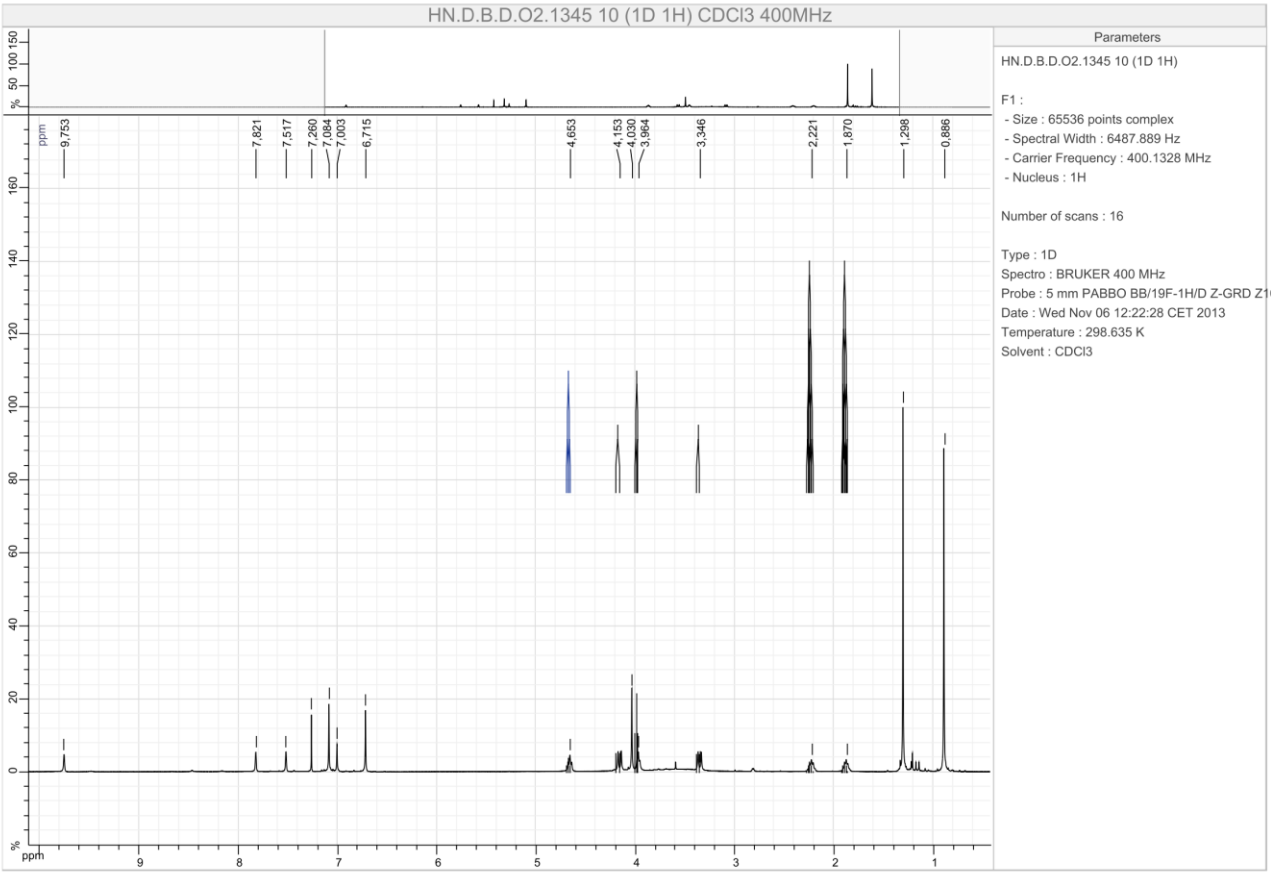

^{1}H NMR spectrum of 4a

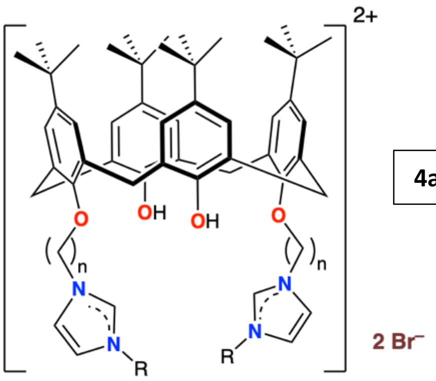

4a, R = Mes, n = 2

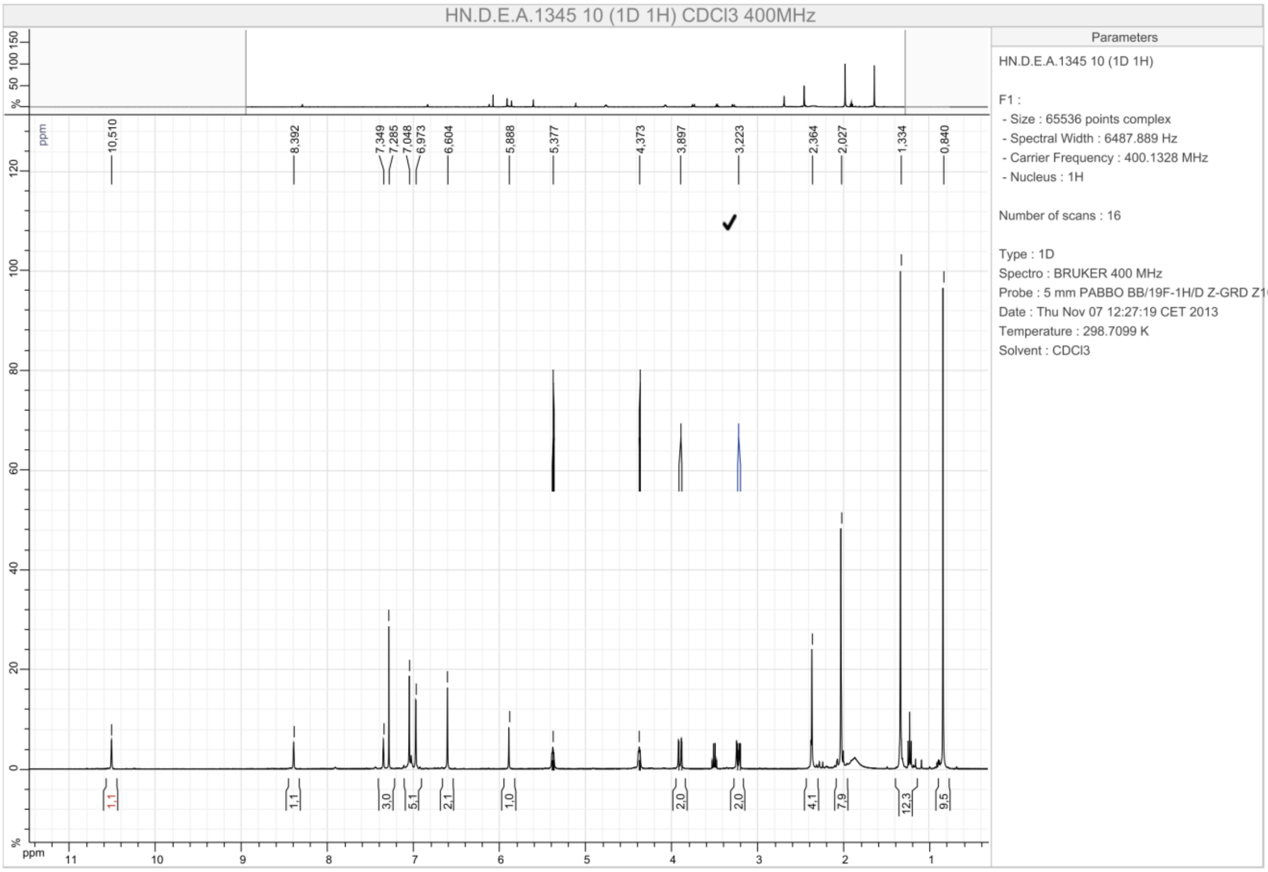

^{1}H NMR spectrum of 4b

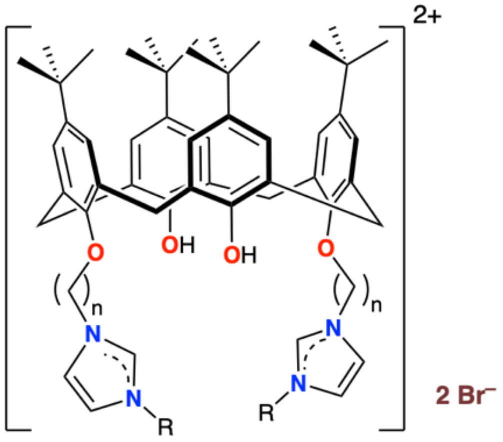

4b, R = Mes, n = 3

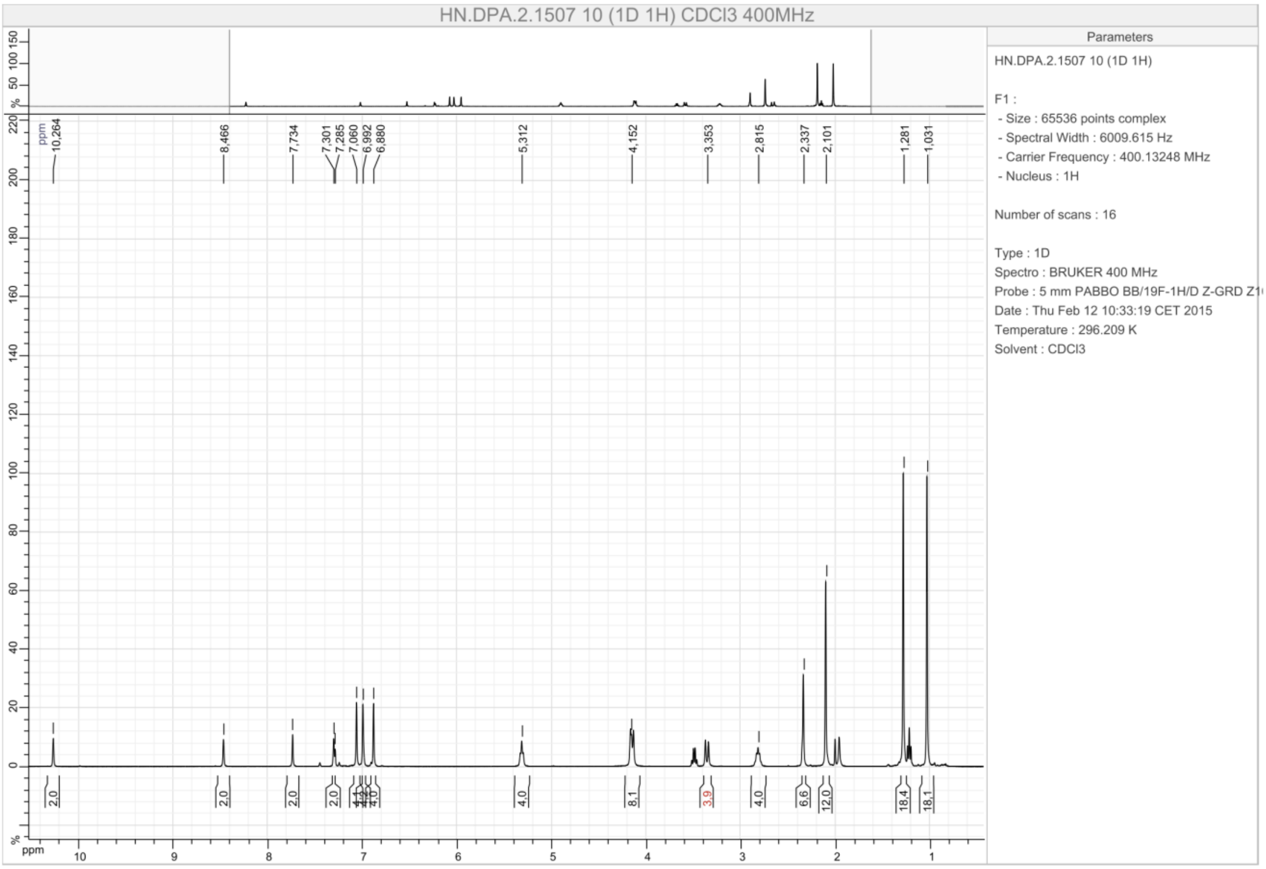

^{1}H NMR spectrum of 4c

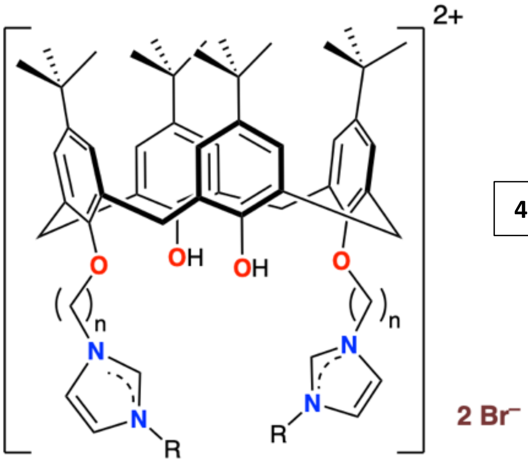

4c, R = Mes, n = 4

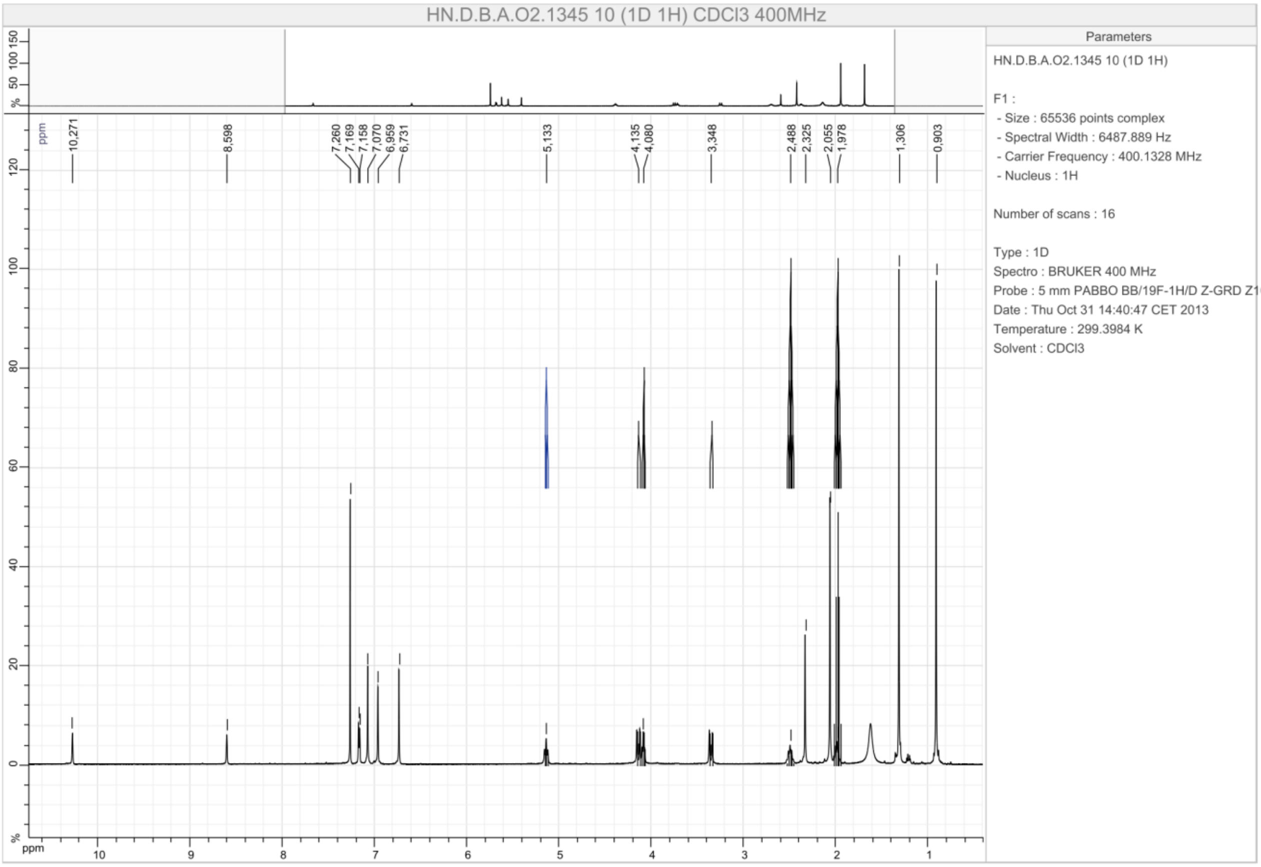

^{1}H NMR spectrum of 5a

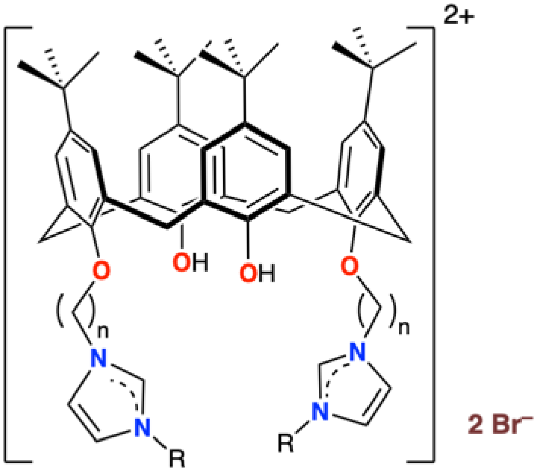

5a, R = DIPP, n = 2

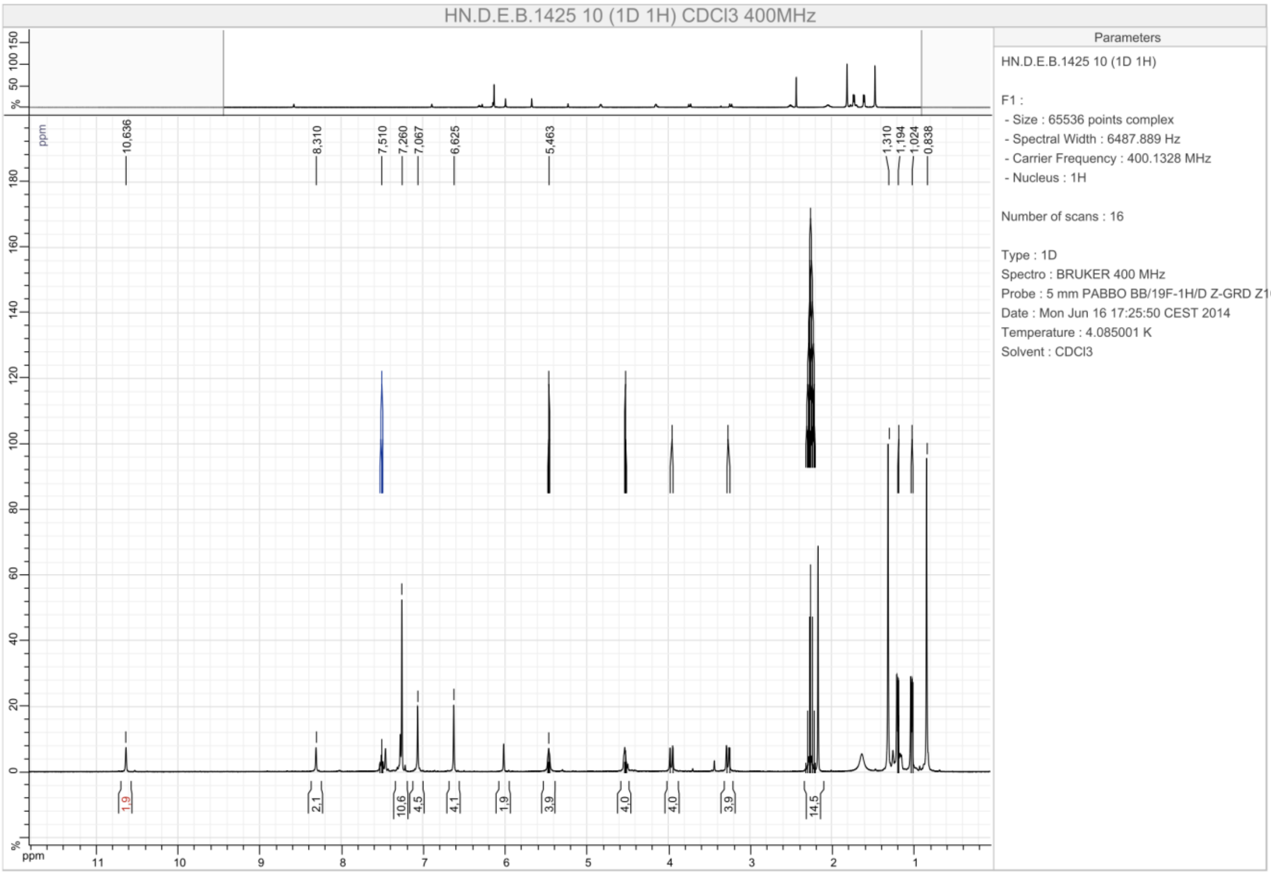

^{1}H NMR spectrum of 5b

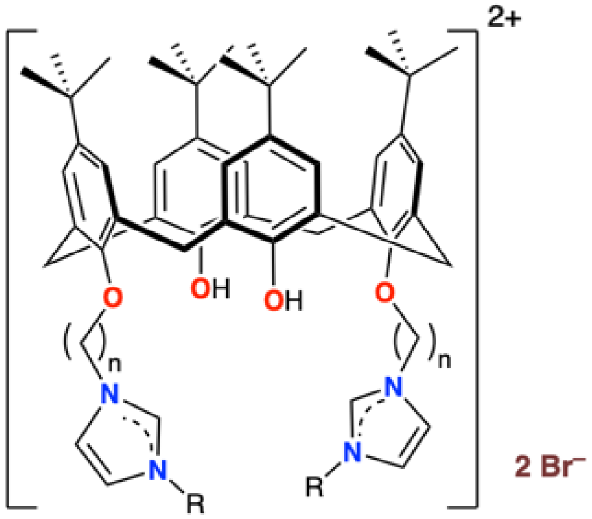

5b, R = DIPP, n = 3

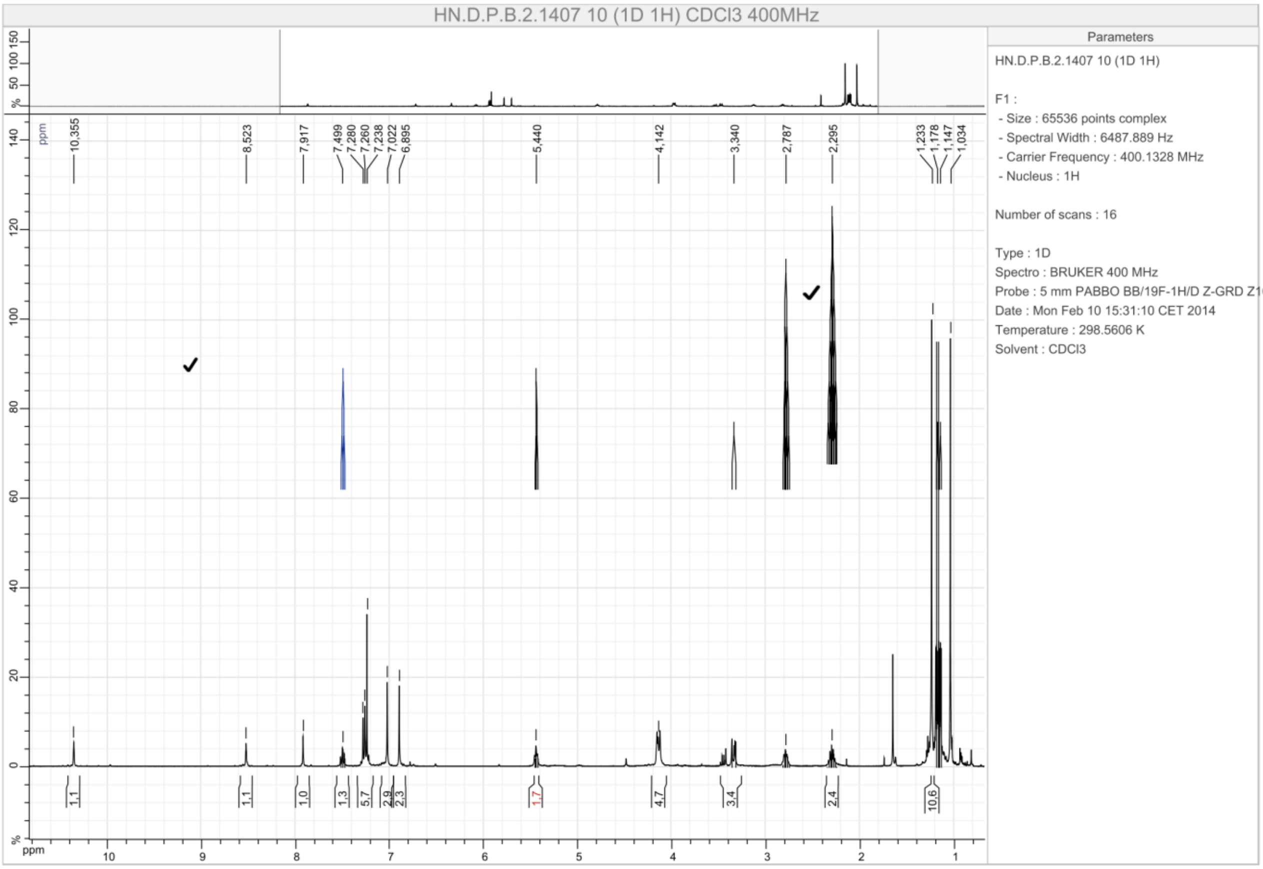

¹H NMR spectrum of 5c

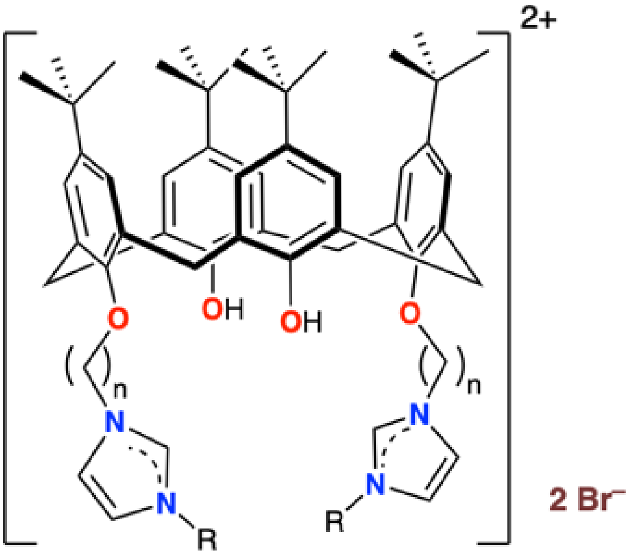

5c, R = DIPP, n = 4

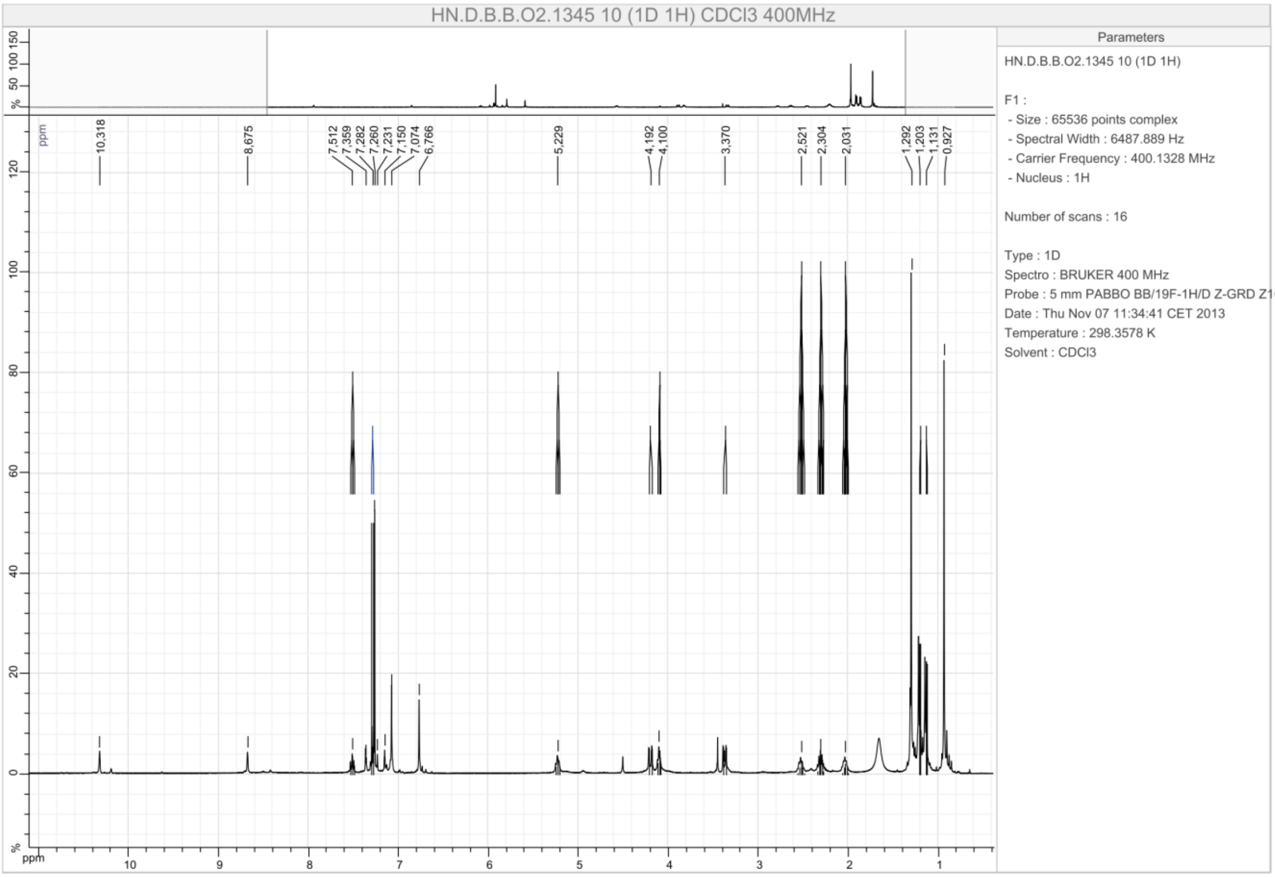
HN.D.B.B.O2.1345 10 (1D 1H) CDCl3 400MHz
10,318
8,675
7,512
7,359
7,282
7,260
7,231
7,150
7,074
6,766
5,229
4,192
4,100
3,370
2,521
2,304
2,031
1,292
1,203
1,131
0,927
Parameters
HN.D.B.B.O2.1345 10 (1D 1H)
F1 :
- Size : 65536 points complex
- Spectral Width : 6487.889 Hz
- Carrier Frequency : 400.1328 MHz
- Nucleus : 1H
Number of scans : 16
Type : 1D
Spectro : BRUKER 400 MHz
Probe : 5 mm PABBO BB/19F-1H/D Z-GRD Z1
Date : Thu Nov 07 11:34:41 CET 2013
Temperature : 298.3578 K
Solvent : CDCl3

^{1}H NMR spectrum of 6a

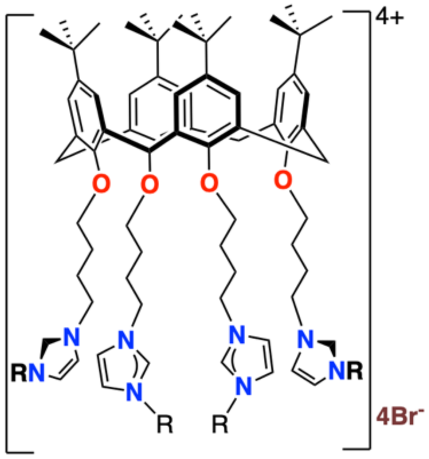

6a, R = Me, n = 4

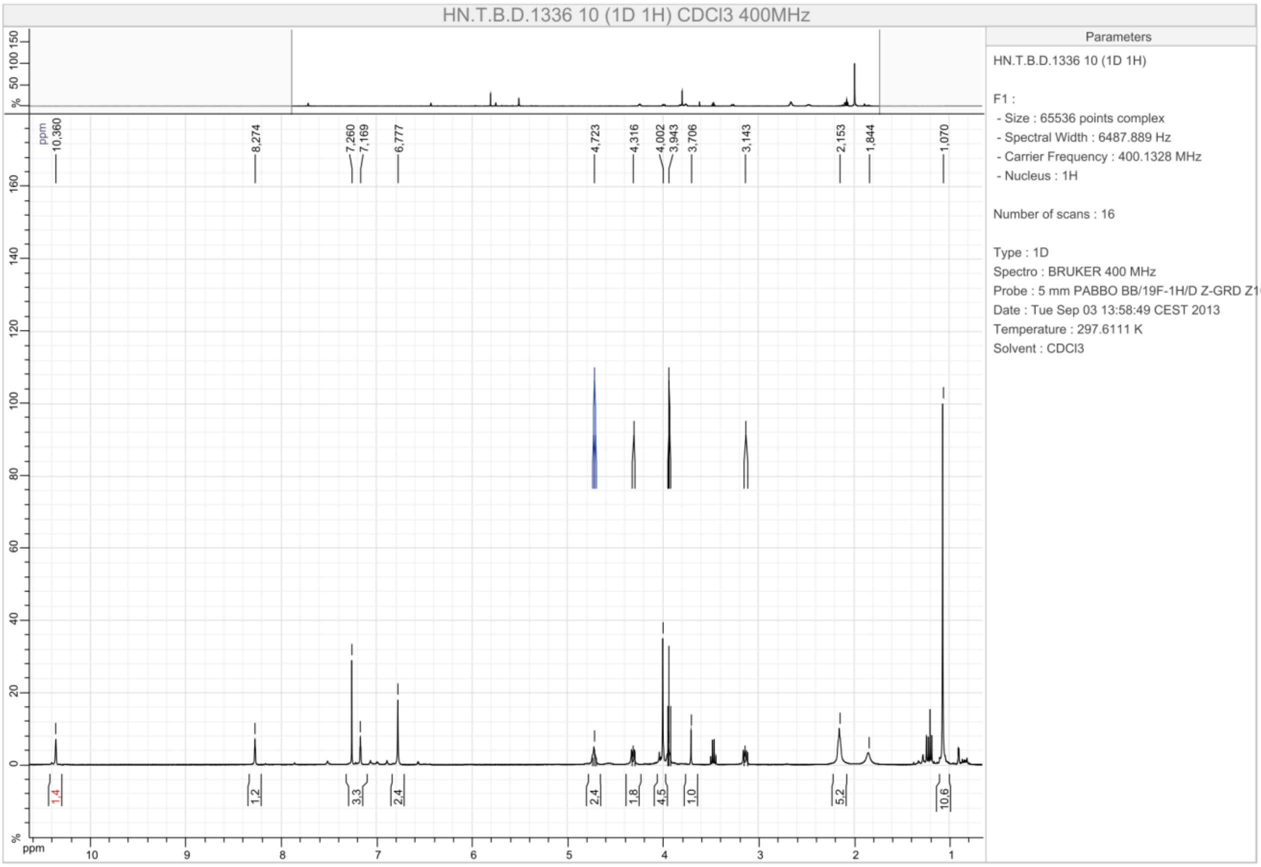

^{1}H NMR spectrum of 6b

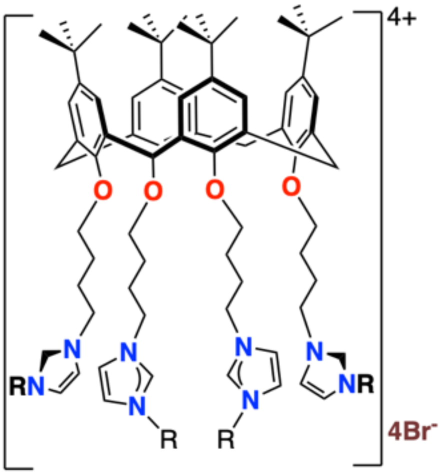

6b, R = Mes, n = 4

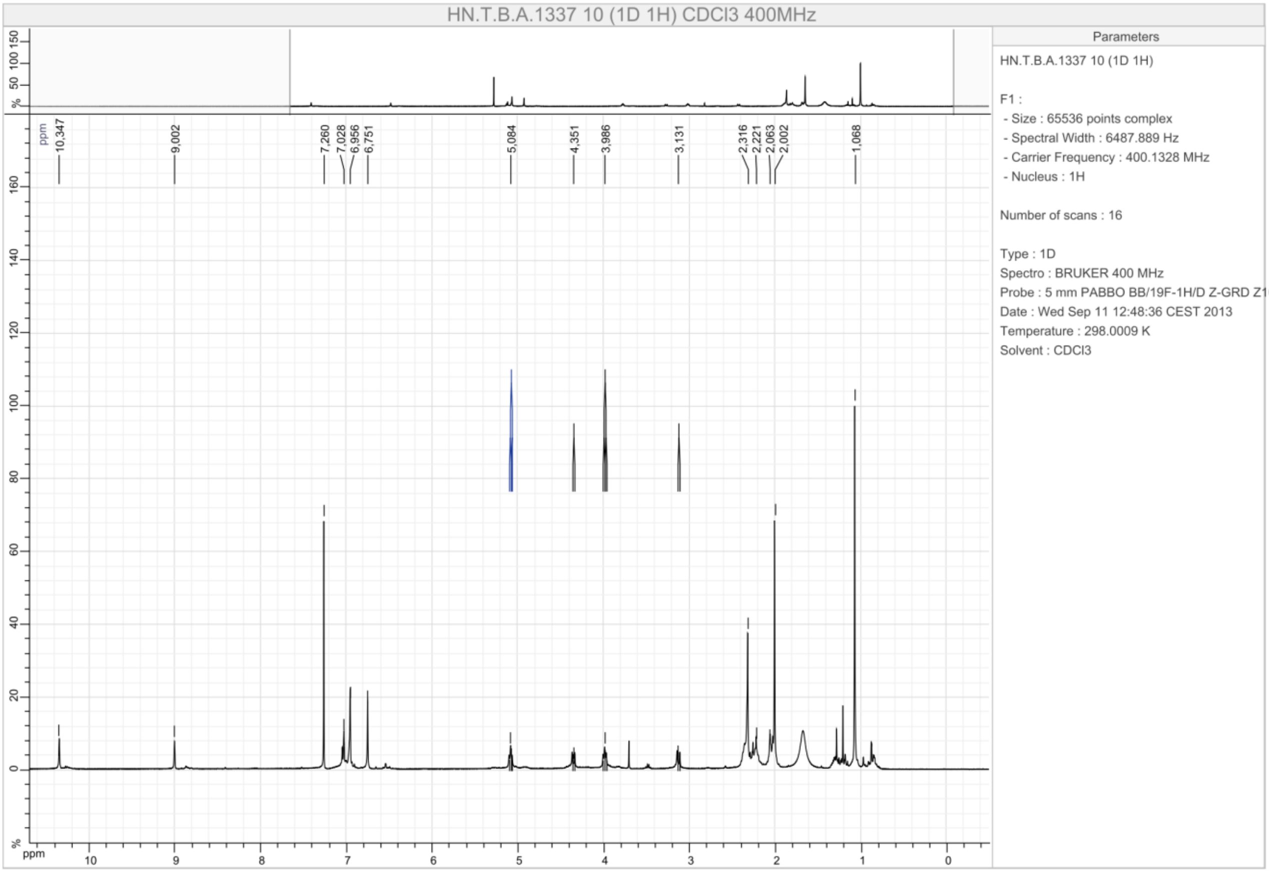

^{1}H NMR spectrum of 6c

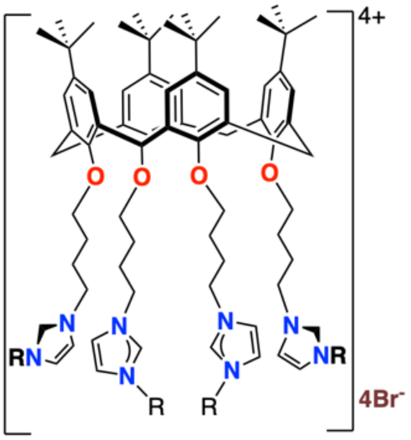

6c, R = DIPP, n

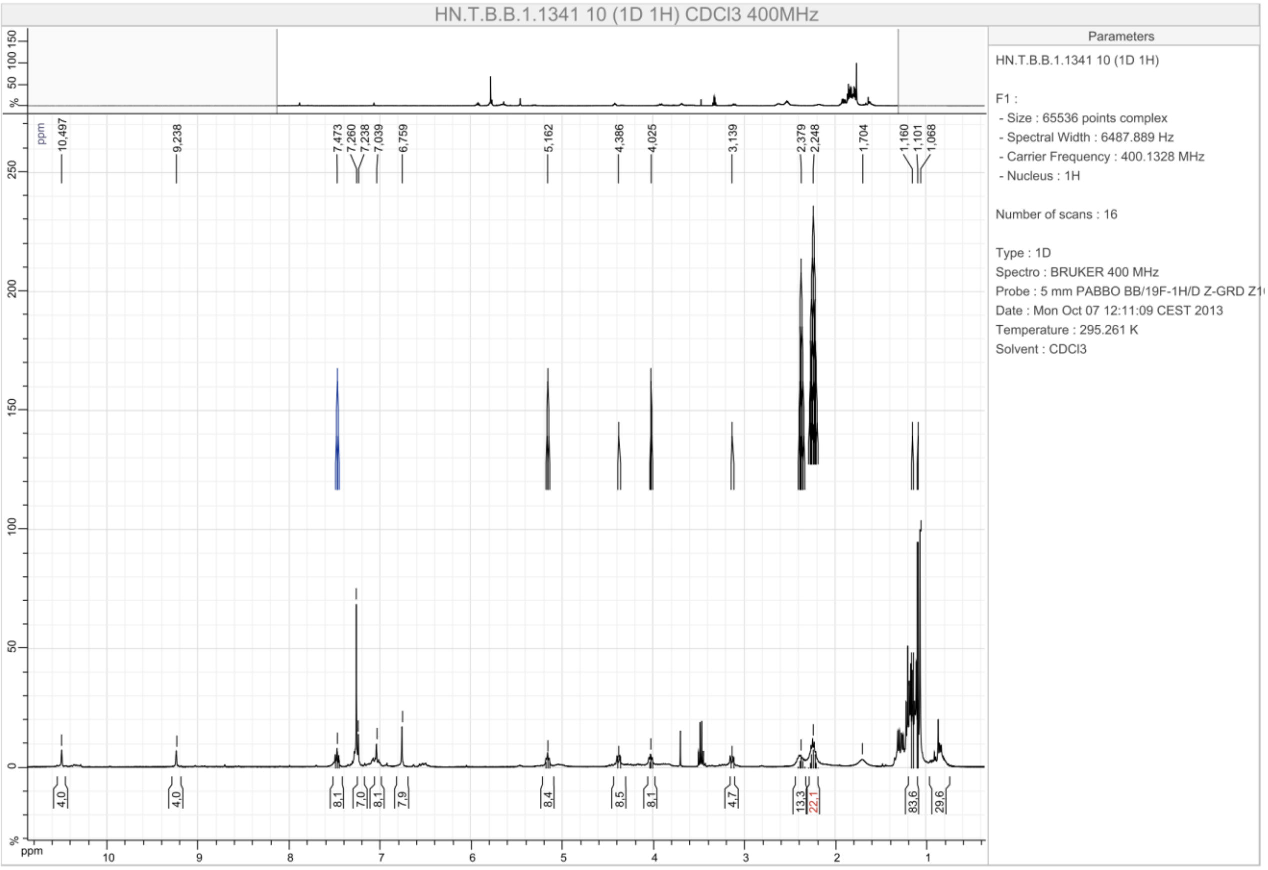

^{1}H NMR spectrum of 7a

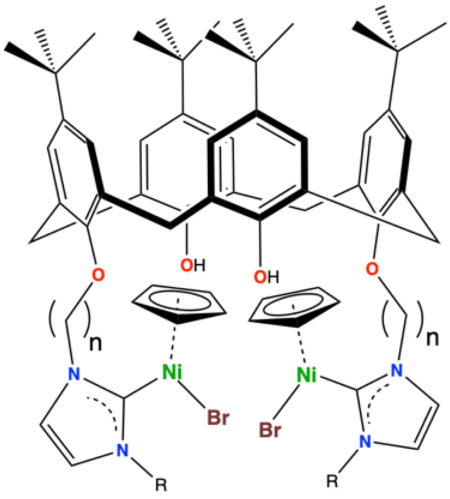

7a, R= Mes, n = 2

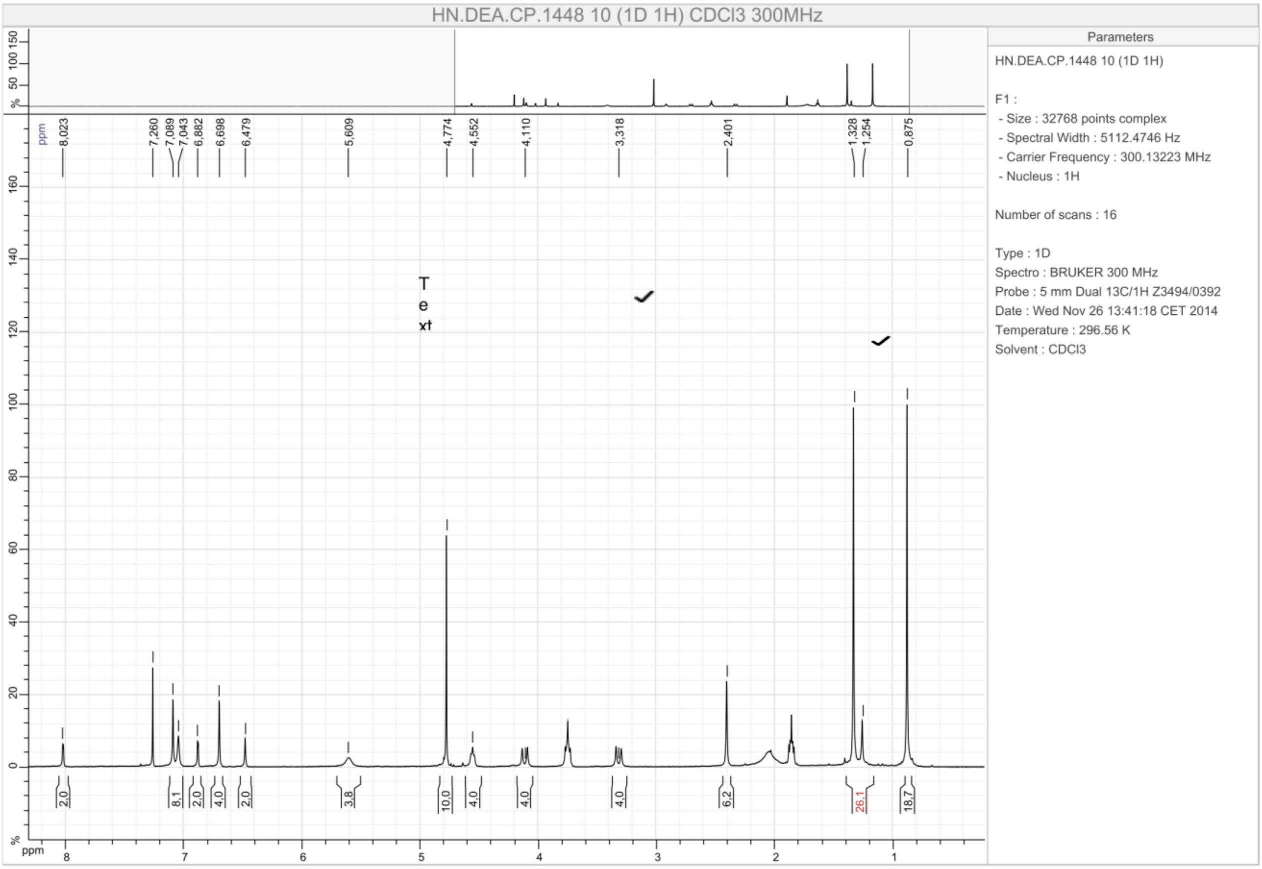

^{1}H NMR spectrum of 7b

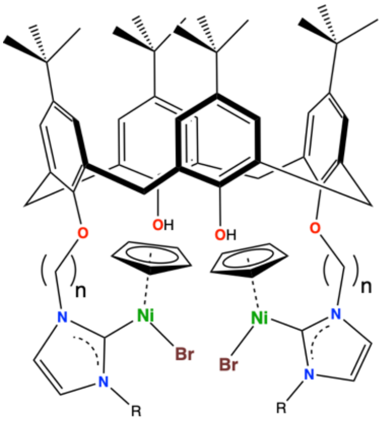

7b, R= Mes, n = 3

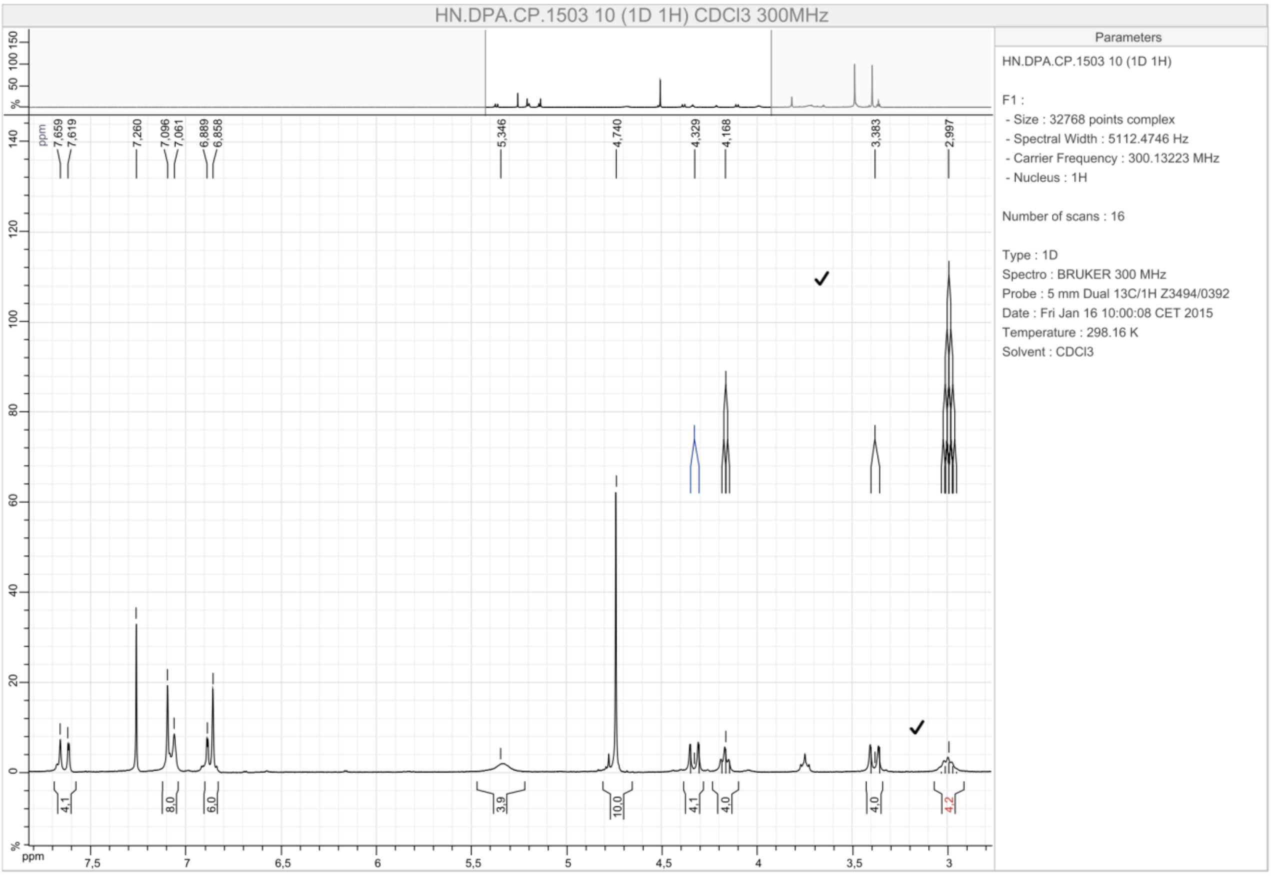

^{1}H NMR spectrum of 7d

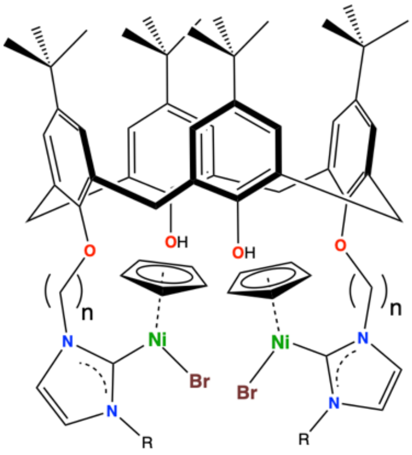

7d, R = DIPP, n = 2

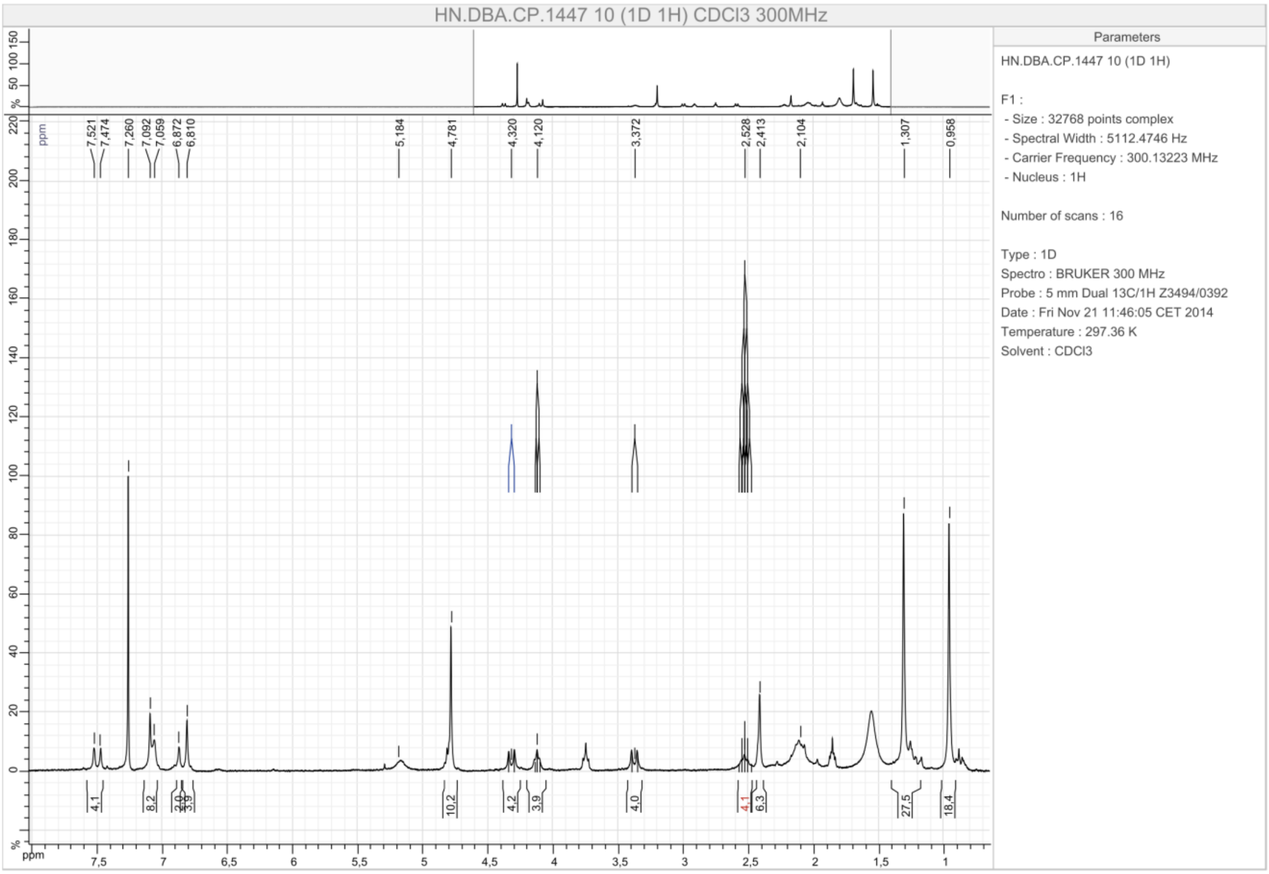

^{13}C NMR spectrum of 3c

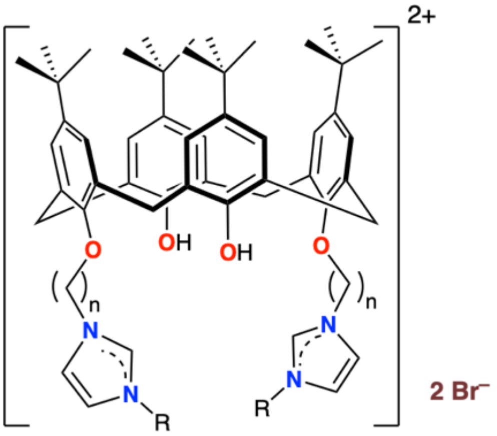

3c, R = Me, n = 4

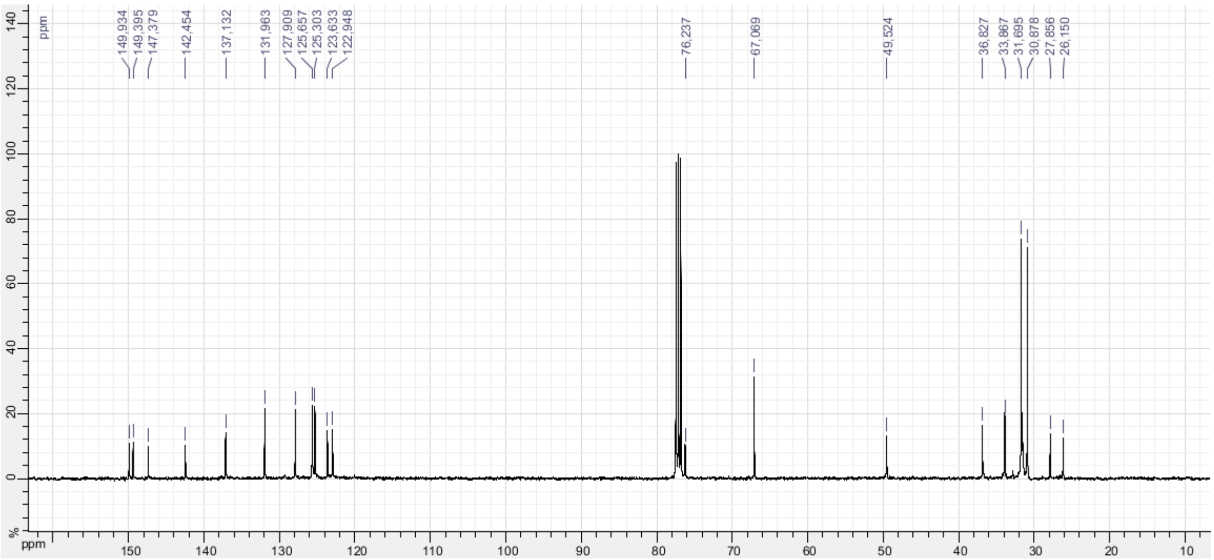
149,934
149,395
147,379
142,454
137,132
131,963
127,909
125,657
125,303
123,633
122,948
76,237
67,069
49,524
36,827
33,867
31,695
30,878
27,856
26,150
ppm
%

^{13}C DEPT NMR spectrum of 3c

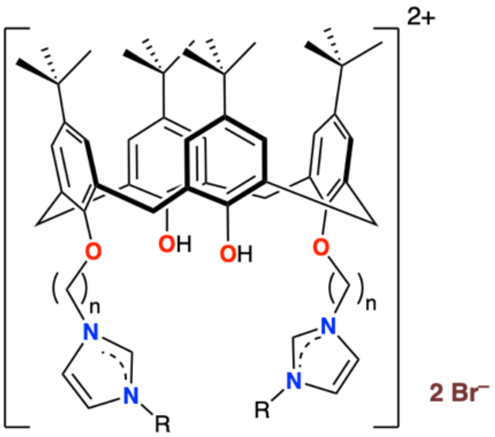

3c, R = Me, n = 4

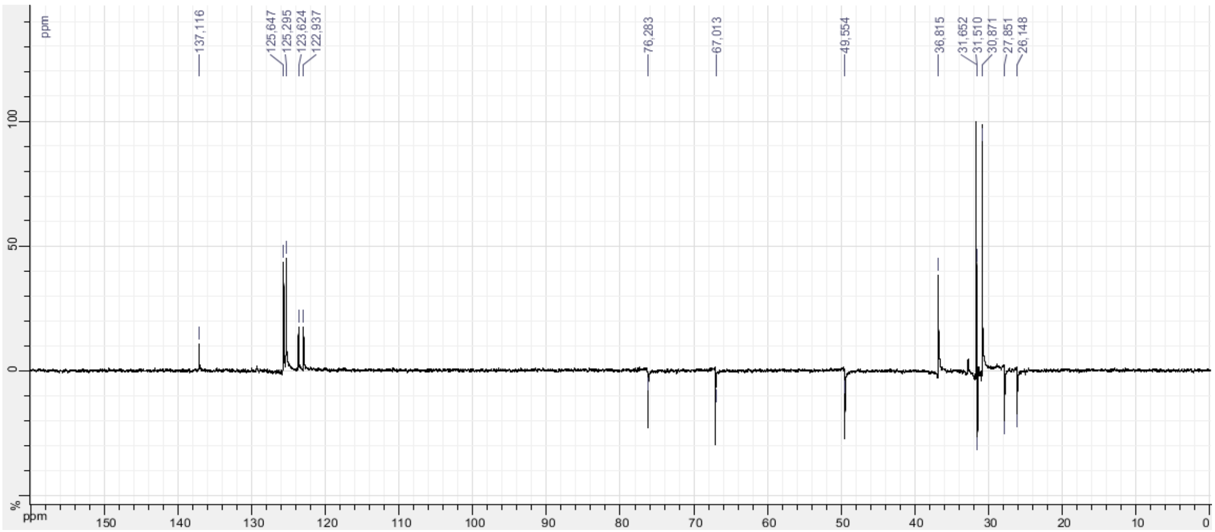

^{13}C NMR spectrum of 4c

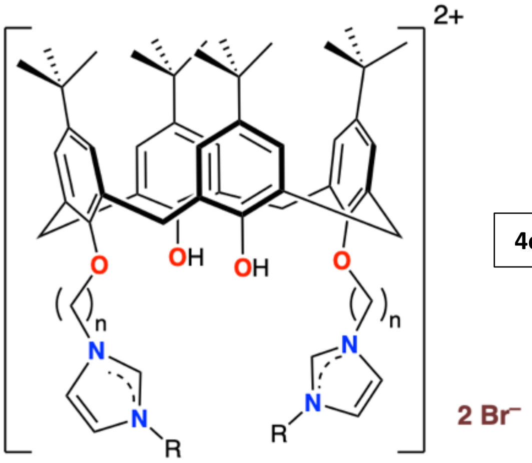

4c, R = Mes, n = 4

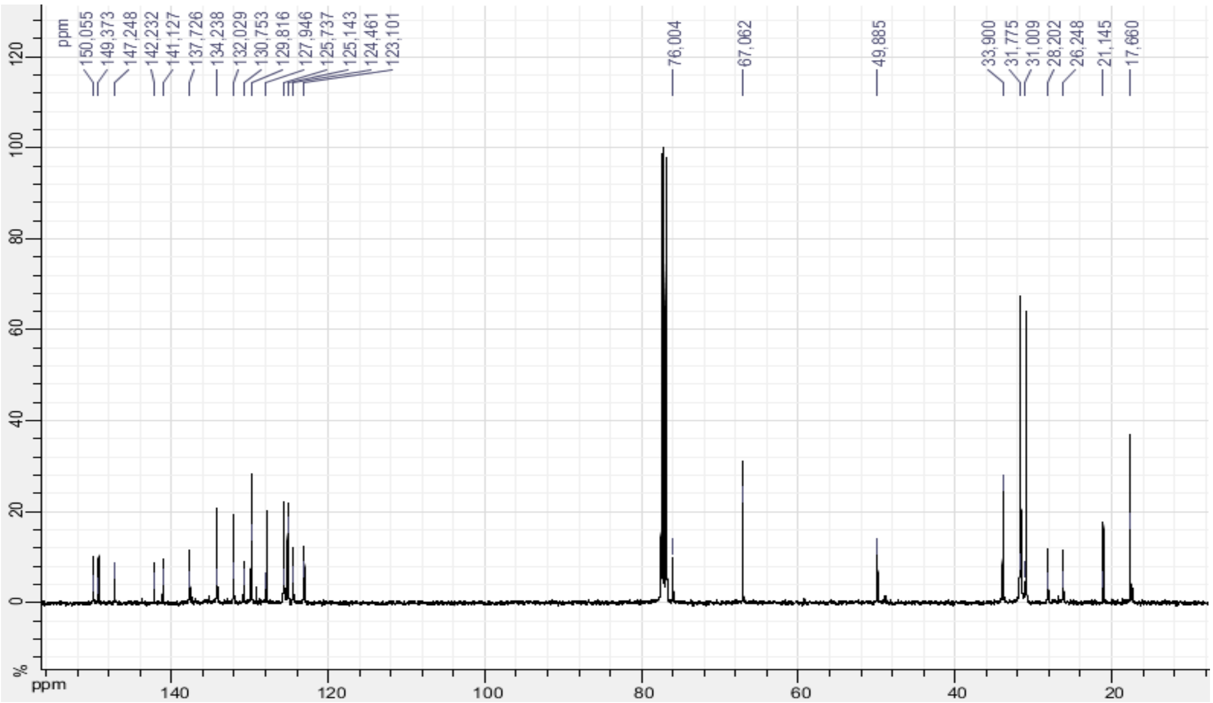

^{13}C DEPT NMR spectrum of 4c

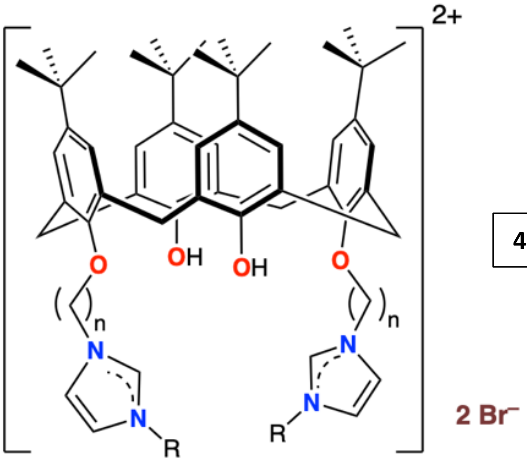

4c, R = Mes, n = 4

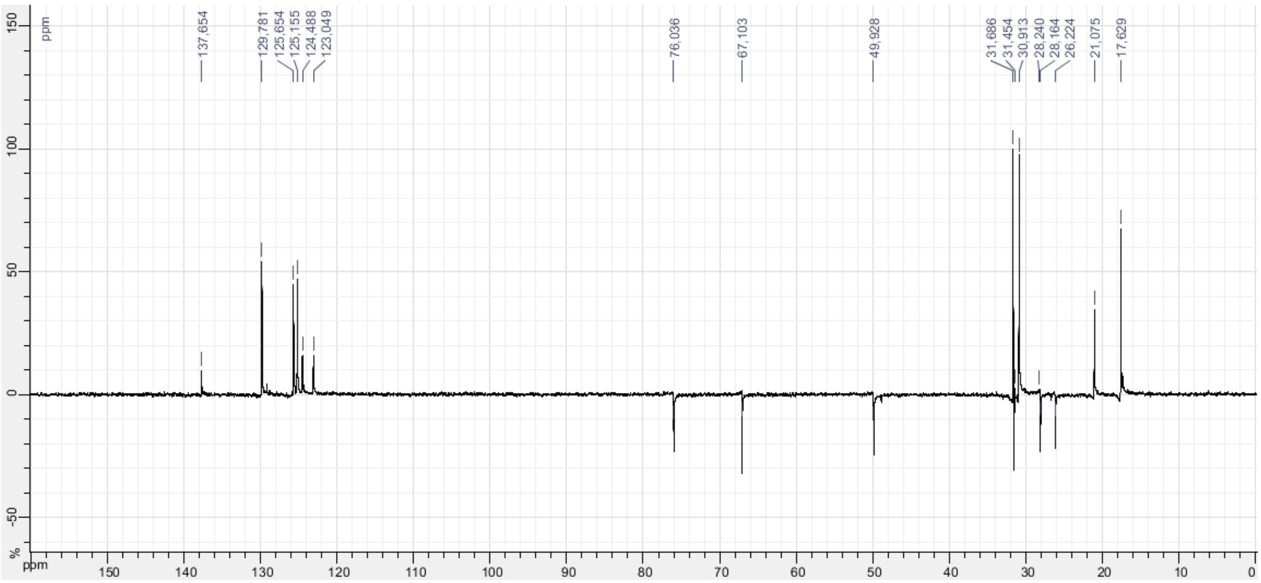

X-ray Data for Complexes 5a and 8 (8 is incomplete and not fully refined)

	**5a**	**8**
Empirical formula	$C_{157}H_{215}Br_3N_8O_{13}$	$C_{76}H_{96}Br_2N_4NiO_4$
Formula weight (g mol^{-1})	2662.09	1348.09
Temperature (K)	173(2)	173(2)
Wavelength (Å)	Mo K 0.71073	Mo K_a 0.71073
Crystal system	Monoclinic	Triclinic
Space group	$P2_1$/c (no. 14)	P_1-bar (no. 2)
a (Å)<br>*b* (Å)<br>*c* (Å)	14.5725(7)<br>31.8666(17)<br>20.4167(8)	16.57(3)<br>18.94(3)<br>19.09(3)
Unit cell angles (°)	90, 116.499 (3), 90	105.61(3), 110, 87(3), 93.53(3)
Volume ($Å^3$)	8485.0(7)	5308(14)
Z	2	2
Density (calcd, Mg/m^3)	1.042	0.844
Abs. coefficient (mm^{-1})	0.767	0.969
F(000)	2844	1420
Crystal size (mm)	0.35 x 0.30 x 0.25	0.25 x 0.15 x 0.05
Data coll. q range (°)	1.592 – 28.034	1.717 – 28.792
Index ranges	-10<h< 19, -42<k<38, -26< *l* <26	-19<h<22, -25<k<23, -25<l<25
Reflections collected	82977	68487
Independent reflections	20460	26519
Completeness to q =	99.5%	95.8%

Absorption correction	Multi scan	Multi scan
Max, min transmission	0.7456, 0.7011	0.5120, 0.7456
Refinement method	Full matrix least squares on F^2	Full matrix least squares on F^2
Data/restraints/parameters	20460/0/851	16519/21/780
Goodness of fit on F^2	1.168	0.1845
Final R indices [$I > 2s(I)$]	R1 = 0.1022, wR2 = 0.3137	R1 = 0.0760, wR2 = 0.1845
R índices (all data)	R1 = 0.1806, wR2 = 0.3610	R1 = 0.2825, wR2 = 0.2774
Largest diff. peak, hole Å^3	1.609, -1.455	

checkCIF/PLATON report

Structure factors have been supplied for datablock(s) mchn141211

THIS REPORT IS FOR GUIDANCE ONLY. IF USED AS PART OF A REVIEW PROCEDURE FOR PUBLICATION, IT SHOULD NOT REPLACE THE EXPERTISE OF AN EXPERIENCED CRYSTALLOGRAPHIC REFEREE.

No syntax errors found. CIF dictionary Interpreting this report

Datablock: mchn141211

Bond precision:	C-C = 0.0076 A		Wavelength=0.71073
Cell:	a=14.5725(7)	b=31.8666(17)	c=20.4167(8)
	alpha=90	beta=116.499(3)	gamma=90
Temperature:	173 K		

	Calculated	Reported
Volume	8485.0(7)	8485.0(7)
Space group	P 21/c	P 21/c
Hall group	-P 2ybc	-P 2ybc
Moiety formula	2(C78 H102 N4 O4), C H3 O, 3(Br), 4(H2 O) [+ solvent]	2(C78 H102 N4 O4), C H3 O, 3(Br), 4(H2 O)
Sum formula	C157 H215 Br3 N8 O13 [+ solvent]	C157 H215 Br3 N8 O13
Mr	2662.08	2662.09
Dx,g cm-3	1.042	1.042
Z	2	2
Mu (mm-1)	0.767	0.767
F000	2844.0	2844.0
F000'	2843.30	
h,k,lmax	19,42,26	19,42,26
Nref	20555	20460
Tmin,Tmax	0.765,0.826	0.701,0.746
Tmin'	0.765	

Correction method= # Reported T Limits: Tmin=0.701 Tmax=0.746
AbsCorr = MULTI-SCAN

Data completeness= 0.995 Theta(max)= 28.034

```
R(reflections)= 0.1022( 10315)                          wR2(reflections)=
                                                        0.3610( 20460)
S = 1.168                      Npar= 851
```

The following ALERTS were generated. Each ALERT has the format
test-name_ALERT_alert-type_alert-level.
Click on the hyperlinks for more details of the test.

Alert level B

```
PLAT084_ALERT_3_B High wR2 Value (i.e. > 0.25) ...................       0.36 Report
PLAT242_ALERT_2_B Low    'MainMol' Ueq as Compared to Neighbors of        C75 Check
PLAT245_ALERT_2_B U(iso) H6W1      Smaller than U(eq) O6        by      0.051 Ang**2
PLAT245_ALERT_2_B U(iso) H6W2      Smaller than U(eq) O6        by      0.051 Ang**2
PLAT412_ALERT_2_B Short Intra XH3 .. XHn     H76C      ..H78A     .       1.72 Ang.
                                                       x,y,z  =      1_555 Check
PLAT420_ALERT_2_B D-H Bond Without Acceptor  O5        --H5W1     .     Please Check
PLAT420_ALERT_2_B D-H Bond Without Acceptor  O5        --H5W2     .     Please Check
PLAT420_ALERT_2_B D-H Bond Without Acceptor  O6        --H6W1     .     Please Check
PLAT420_ALERT_2_B D-H Bond Without Acceptor  O6        --H6W2     .     Please Check
```

Alert level C

```
PLAT213_ALERT_2_C Atom C77             has ADP max/min Ratio .....        3.7 prolat
PLAT213_ALERT_2_C Atom C78             has ADP max/min Ratio .....        3.1 prolat
PLAT214_ALERT_2_C Atom O6        (Anion/Solvent) ADP max/min Ratio        4.2 prolat
PLAT220_ALERT_2_C NonSolvent   Resd 1  C   Ueq(max)/Ueq(min) Range        5.9 Ratio
PLAT222_ALERT_3_C NonSolvent Resd 1  H   Uiso(max)/Uiso(min) Range        6.6 Ratio
PLAT241_ALERT_2_C High   'MainMol' Ueq as Compared to Neighbors of        C36 Check
PLAT241_ALERT_2_C High   'MainMol' Ueq as Compared to Neighbors of        C37 Check
PLAT242_ALERT_2_C Low    'MainMol' Ueq as Compared to Neighbors of         N1 Check
PLAT242_ALERT_2_C Low    'MainMol' Ueq as Compared to Neighbors of         N2 Check
PLAT242_ALERT_2_C Low    'MainMol' Ueq as Compared to Neighbors of        C29 Check
PLAT242_ALERT_2_C Low    'MainMol' Ueq as Compared to Neighbors of        C44 Check
PLAT242_ALERT_2_C Low    'MainMol' Ueq as Compared to Neighbors of        C47 Check
PLAT242_ALERT_2_C Low    'MainMol' Ueq as Compared to Neighbors of        C54 Check
PLAT242_ALERT_2_C Low    'MainMol' Ueq as Compared to Neighbors of        C69 Check
PLAT242_ALERT_2_C Low    'MainMol' Ueq as Compared to Neighbors of        C72 Check
PLAT245_ALERT_2_C U(iso) H5W1      Smaller than U(eq) O5        by      0.014 Ang**2
PLAT245_ALERT_2_C U(iso) H5W2      Smaller than U(eq) O5        by      0.014 Ang**2
PLAT260_ALERT_2_C Large Average Ueq of Residue Including        O8      0.109 Check
PLAT260_ALERT_2_C Large Average Ueq of Residue Including        O6      0.101 Check
PLAT309_ALERT_2_C Single Bonded Oxygen (C-O > 1.3 Ang) ...........         O8 Check
PLAT341_ALERT_3_C Low Bond Precision on  C-C Bonds ...............     0.0076 Ang.
PLAT354_ALERT_3_C Short  O-H (X0.82,N0.98A)  O5        - H5W1     .       0.64 Ang.
PLAT360_ALERT_2_C Short  C(sp3)-C(sp3) Bond  C75       - C77      .       1.42 Ang.
PLAT360_ALERT_2_C Short  C(sp3)-C(sp3) Bond  C75       - C78      .       1.41 Ang.
PLAT412_ALERT_2_C Short Intra XH3 .. XHn     H23       ..H78B     .       1.87 Ang.
                                                       x,y,z  =      1_555 Check
PLAT412_ALERT_2_C Short Intra XH3 .. XHn     H76A      ..H77B     .       1.86 Ang.
                                                       x,y,z  =      1_555 Check
PLAT905_ALERT_3_C Negative K value in the Analysis of Variance ...     -1.503 Report
PLAT911_ALERT_3_C Missing FCF Refl Between Thmin & STh/L=    0.600         22 Report
PLAT918_ALERT_3_C Reflection(s) with I(obs) much Smaller I(calc) .         17 Check
PLAT934_ALERT_3_C Number of (Iobs-Icalc)/Sigma(W) > 10 Outliers ..          1 Check
```

```
PLAT971_ALERT_2_C Check Calcd Resid. Dens.  1.51Ang From Br1              1.52 eA-3
PLAT975_ALERT_2_C Check Calcd Resid. Dens.  1.10Ang From O5      .        1.14 eA-3
PLAT975_ALERT_2_C Check Calcd Resid. Dens.  0.76Ang From O6      .        0.99 eA-3
PLAT975_ALERT_2_C Check Calcd Resid. Dens.  0.49Ang From O5      .        0.96 eA-3
PLAT975_ALERT_2_C Check Calcd Resid. Dens.  0.91Ang From O5      .        0.94 eA-3
PLAT975_ALERT_2_C Check Calcd Resid. Dens.  0.85Ang From O5      .        0.94 eA-3
PLAT975_ALERT_2_C Check Calcd Resid. Dens.  1.04Ang From O6      .        0.93 eA-3
PLAT975_ALERT_2_C Check Calcd Resid. Dens.  0.51Ang From O5      .        0.86 eA-3
PLAT975_ALERT_2_C Check Calcd Resid. Dens.  0.83Ang From O6      .        0.79 eA-3
```

Alert level G

```
PLAT007_ALERT_5_G Number of Unrefined Donor-H Atoms ..............          6 Report
PLAT128_ALERT_4_G Alternate Setting for Input Space Group  P21/c       P21/n Note
PLAT169_ALERT_4_G The CIF-Embedded .res File Contains AFIX 1 Recds          1 Report
PLAT171_ALERT_4_G The CIF-Embedded .res File Contains EADP Records          1 Report
PLAT230_ALERT_2_G Hirshfeld Test Diff for    C54      --C55      .        6.2 s.u.
PLAT230_ALERT_2_G Hirshfeld Test Diff for    C54      --C55B     .        5.3 s.u.
PLAT230_ALERT_2_G Hirshfeld Test Diff for    C54      --C56      .        7.3 s.u.
PLAT230_ALERT_2_G Hirshfeld Test Diff for    C54      --C56B     .        6.0 s.u.
PLAT230_ALERT_2_G Hirshfeld Test Diff for    C54      --C57      .        6.2 s.u.
PLAT230_ALERT_2_G Hirshfeld Test Diff for    C54      --C57B     .        6.2 s.u.
PLAT300_ALERT_4_G Atom Site Occupancy of C55        Constrained at        0.5 Check
PLAT300_ALERT_4_G Atom Site Occupancy of C55B       Constrained at        0.5 Check
PLAT300_ALERT_4_G Atom Site Occupancy of C56        Constrained at        0.5 Check
PLAT300_ALERT_4_G Atom Site Occupancy of C56B       Constrained at        0.5 Check
PLAT300_ALERT_4_G Atom Site Occupancy of C57        Constrained at        0.5 Check
PLAT300_ALERT_4_G Atom Site Occupancy of C57B       Constrained at        0.5 Check
PLAT300_ALERT_4_G Atom Site Occupancy of H55A       Constrained at        0.5 Check
PLAT300_ALERT_4_G Atom Site Occupancy of H55B       Constrained at        0.5 Check
PLAT300_ALERT_4_G Atom Site Occupancy of H55C       Constrained at        0.5 Check
PLAT300_ALERT_4_G Atom Site Occupancy of H55D       Constrained at        0.5 Check
PLAT300_ALERT_4_G Atom Site Occupancy of H55E       Constrained at        0.5 Check
PLAT300_ALERT_4_G Atom Site Occupancy of H55F       Constrained at        0.5 Check
PLAT300_ALERT_4_G Atom Site Occupancy of H56A       Constrained at        0.5 Check
PLAT300_ALERT_4_G Atom Site Occupancy of H56B       Constrained at        0.5 Check
PLAT300_ALERT_4_G Atom Site Occupancy of H56C       Constrained at        0.5 Check
PLAT300_ALERT_4_G Atom Site Occupancy of H56D       Constrained at        0.5 Check
PLAT300_ALERT_4_G Atom Site Occupancy of H56E       Constrained at        0.5 Check
PLAT300_ALERT_4_G Atom Site Occupancy of H56F       Constrained at        0.5 Check
PLAT300_ALERT_4_G Atom Site Occupancy of H57A       Constrained at        0.5 Check
PLAT300_ALERT_4_G Atom Site Occupancy of H57B       Constrained at        0.5 Check
PLAT300_ALERT_4_G Atom Site Occupancy of H57C       Constrained at        0.5 Check
PLAT300_ALERT_4_G Atom Site Occupancy of H57D       Constrained at        0.5 Check
PLAT300_ALERT_4_G Atom Site Occupancy of H57E       Constrained at        0.5 Check
PLAT300_ALERT_4_G Atom Site Occupancy of H57F       Constrained at        0.5 Check
PLAT300_ALERT_4_G Atom Site Occupancy of O8         Constrained at        0.5 Check
PLAT300_ALERT_4_G Atom Site Occupancy of C80        Constrained at        0.5 Check
PLAT300_ALERT_4_G Atom Site Occupancy of H80A       Constrained at        0.5 Check
PLAT300_ALERT_4_G Atom Site Occupancy of H80B       Constrained at        0.5 Check
PLAT300_ALERT_4_G Atom Site Occupancy of H80C       Constrained at        0.5 Check
PLAT300_ALERT_4_G Atom Site Occupancy of Br2        Constrained at        0.5 Check
PLAT301_ALERT_3_G Main Residue  Disorder ..............(Resd  1  )         3% Note
PLAT302_ALERT_4_G Anion/Solvent/Minor-Residue Disorder (Resd  2  )       100% Note
PLAT302_ALERT_4_G Anion/Solvent/Minor-Residue Disorder (Resd  4  )       100% Note
PLAT304_ALERT_4_G Non-Integer Number of Atoms in ..... (Resd  2  )       2.50 Check
PLAT304_ALERT_4_G Non-Integer Number of Atoms in ..... (Resd  4  )       0.50 Check
```

```
PLAT412_ALERT_2_G Short Intra XH3 .. XHn     H16      ..H55C     .       1.91 Ang.
                                                      x,y,z  =      1_555 Check
PLAT412_ALERT_2_G Short Intra XH3 .. XHn     H16      ..H55E     .       1.99 Ang.
                                                      x,y,z  =      1_555 Check
PLAT412_ALERT_2_G Short Intra XH3 .. XHn     H18      ..H56B     .       2.11 Ang.
                                                      x,y,z  =      1_555 Check
PLAT606_ALERT_4_G Solvent Accessible VOID(S) in Structure ........          ! Info
PLAT720_ALERT_4_G Number of Unusual/Non-Standard Labels ..........          4 Note
PLAT869_ALERT_4_G ALERTS Related to the Use of SQUEEZE  Suppressed          ! Info
PLAT910_ALERT_3_G Missing # of FCF Reflection(s) Below Theta(Min).          3 Note
PLAT912_ALERT_4_G Missing # of FCF Reflections Above STh/L=  0.600         69 Note
PLAT933_ALERT_2_G Number of HKL-OMIT Records in Embedded .res File         17 Note
PLAT941_ALERT_3_G Average HKL Measurement Multiplicity ...........        4.1 Low
PLAT978_ALERT_2_G Number C-C Bonds with Positive Residual Density.          0 Info
```

```
   0 ALERT level A = Most likely a serious problem - resolve or explain
   9 ALERT level B = A potentially serious problem, consider carefully
  39 ALERT level C = Check. Ensure it is not caused by an omission or oversight
  56 ALERT level G = General information/check it is not something unexpected

   0 ALERT type 1 CIF construction/syntax error, inconsistent or missing data
  51 ALERT type 2 Indicator that the structure model may be wrong or deficient
  11 ALERT type 3 Indicator that the structure quality may be low
  41 ALERT type 4 Improvement, methodology, query or suggestion
   1 ALERT type 5 Informative message, check
```

Validation response form

Please find below a validation response form (VRF) that can be filled in and pasted into your CIF.

```
# start Validation Reply Form
_vrf_PLAT084_mchn141211
;
PROBLEM: High wR2 Value (i.e. > 0.25) ...................       0.36 Report
RESPONSE: ...
;
_vrf_PLAT242_mchn141211
;
PROBLEM: Low    'MainMol' Ueq as Compared to Neighbors of        C75 Check
RESPONSE: ...
;
_vrf_PLAT245_mchn141211
;
PROBLEM: U(iso) H6W1      Smaller than U(eq) O6        by      0.051 Ang**2
RESPONSE: ...
;
_vrf_PLAT412_mchn141211
;
PROBLEM: Short Intra XH3 .. XHn     H76C     ..H78A     .       1.72 Ang.
RESPONSE: ...
;
_vrf_PLAT420_mchn141211
;
PROBLEM: D-H Bond Without Acceptor  O5       --H5W1     .     Please Check
RESPONSE: ...
```

```
;
_vrf_PLAT213_mchn141211
;
PROBLEM: Atom C77             has ADP max/min Ratio .....         3.7 prolat
RESPONSE: ...
;
_vrf_PLAT214_mchn141211
;
PROBLEM: Atom O6        (Anion/Solvent) ADP max/min Ratio         4.2 prolat
RESPONSE: ...
;
_vrf_PLAT220_mchn141211
;
PROBLEM: NonSolvent   Resd 1  C   Ueq(max)/Ueq(min) Range         5.9 Ratio
RESPONSE: ...
;
_vrf_PLAT222_mchn141211
;
PROBLEM: NonSolvent Resd 1  H   Uiso(max)/Uiso(min) Range         6.6 Ratio
RESPONSE: ...
;
_vrf_PLAT241_mchn141211
;
PROBLEM: High   'MainMol' Ueq as Compared to Neighbors of         C36 Check
RESPONSE: ...
;
_vrf_PLAT260_mchn141211
;
PROBLEM: Large Average Ueq of Residue Including        O8       0.109 Check
RESPONSE: ...
;
_vrf_PLAT309_mchn141211
;
PROBLEM: Single Bonded Oxygen (C-O > 1.3 Ang) ...........          O8 Check
RESPONSE: ...
;
_vrf_PLAT341_mchn141211
;
PROBLEM: Low Bond Precision on  C-C Bonds ...............      0.0076 Ang.
RESPONSE: ...
;
_vrf_PLAT354_mchn141211
;
PROBLEM: Short  O-H (X0.82,N0.98A)  O5       - H5W1     .        0.64 Ang.
RESPONSE: ...
;
_vrf_PLAT360_mchn141211
;
PROBLEM: Short  C(sp3)-C(sp3) Bond  C75      - C77      .        1.42 Ang.
RESPONSE: ...
;
_vrf_PLAT905_mchn141211
;
PROBLEM: Negative K value in the Analysis of Variance ...      -1.503 Report
RESPONSE: ...
;
_vrf_PLAT911_mchn141211
```

```
;
PROBLEM: Missing FCF Refl Between Thmin & STh/L=    0.600          22 Report
RESPONSE: ...
;
_vrf_PLAT918_mchn141211
;
PROBLEM: Reflection(s) with I(obs) much Smaller I(calc) .         17 Check
RESPONSE: ...
;
_vrf_PLAT934_mchn141211
;
PROBLEM: Number of (Iobs-Icalc)/Sigma(W) > 10 Outliers ..          1 Check
RESPONSE: ...
;
_vrf_PLAT971_mchn141211
;
PROBLEM: Check Calcd Resid. Dens.  1.51Ang From Br1            1.52 eA-3
RESPONSE: ...
;
_vrf_PLAT975_mchn141211
;
PROBLEM: Check Calcd Resid. Dens.  1.10Ang From O5      .       1.14 eA-3
RESPONSE: ...
;
# end Validation Reply Form
```

It is advisable to attempt to resolve as many as possible of the alerts in all categories. Often the minor alerts point to easily fixed oversights, errors and omissions in your CIF or refinement strategy, so attention to these fine details can be worthwhile. In order to resolve some of the more serious problems it may be necessary to carry out additional measurements or structure refinements. However, the purpose of your study may justify the reported deviations and the more serious of these should normally be commented upon in the discussion or experimental section of a paper or in the "special_details" fields of the CIF. checkCIF was carefully designed to identify outliers and unusual parameters, but every test has its limitations and alerts that are not important in a particular case may appear. Conversely, the absence of alerts does not guarantee there are no aspects of the results needing attention. It is up to the individual to critically assess their own results and, if necessary, seek expert advice.

Publication of your CIF in IUCr journals

A basic structural check has been run on your CIF. These basic checks will be run on all CIFs submitted for publication in IUCr journals (*Acta Crystallographica*, *Journal of Applied Crystallography*, *Journal of Synchrotron Radiation*); however, if you intend to submit to *Acta Crystallographica Section C* or *E* or *IUCrData*, you should make sure that full publication checks are run on the final version of your CIF prior to submission.

Publication of your CIF in other journals

Please refer to the *Notes for Authors* of the relevant journal for any special instructions relating to CIF submission.

PLATON version of 28/11/2022; check.def file version of 28/11/2022

Datablock mchn141211 - ellipsoid plot

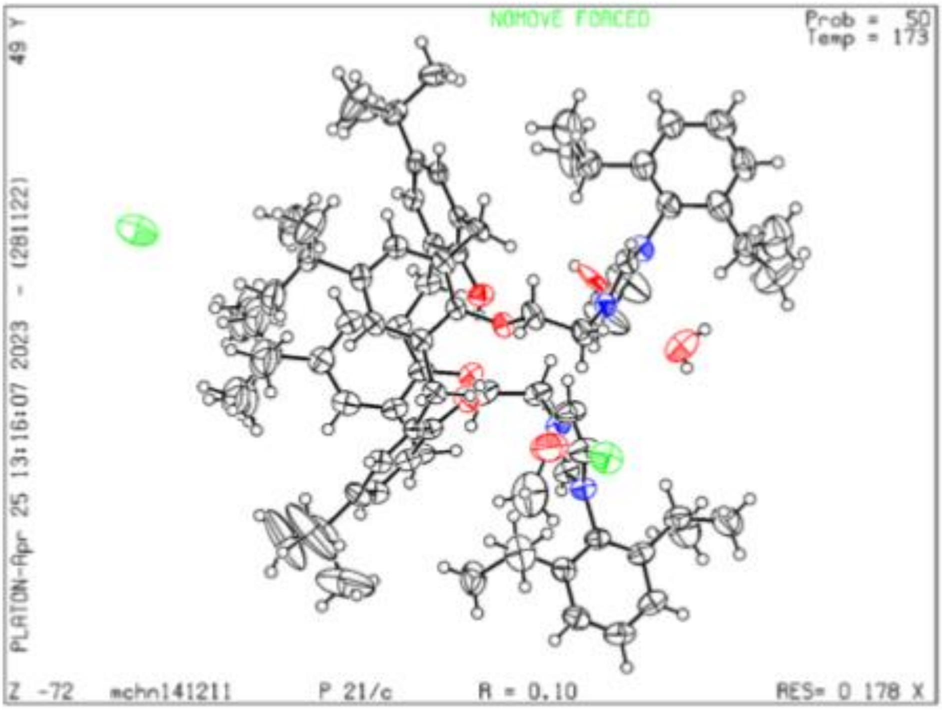

Supplement: Supplementary file 1 [file molecules-28-05697-s001.zip › molecules-2456778-supplementary.pdf]
